# Not just neurons: glial mechanisms driving sex-specific vulnerability to withdrawal and relapse in substance use disorder

**DOI:** 10.1016/j.yfrne.2026.101236

**Published:** 2026-02-12

**Authors:** Nicole Horan, Tori Keefauver, Marianne L. Seney

**Affiliations:** a Department of Psychiatry, University of Pittsburgh School of Medicine, Pittsburgh, PA, USA; b Center for Neuroscience at University of Pittsburgh, Pittsburgh, PA, USA

**Keywords:** Glia, Astrocytes, Microglia, Oligodendrocytes, Sex differences, Withdrawal, Substance use disorder, Nucleus accumbens, Amygdala, Cortex

## Abstract

Sex differences are fundamental determinants of the neurobiology of substance use disorders (SUDs), modulating withdrawal severity, relapse risk, and treatment response. Despite this, prior research has largely studied males and ignored sex differences. Growing bodies of evidence suggest glial cells mediate these sex differences, particularly during withdrawal. In this review, we begin describing baseline sex differences and known glial sex differences at homeostasis. We then examine clinical and preclinical findings describing alterations in glial cells during withdrawal. We describe how glial sex differences are region-specific to areas implicated in reward-induced dysfunction. We conclude with a description of how glial sex differences during withdrawal likely drive sex differences in relapse vulnerability. Our synthesis posits glial cells as key mediators of sex differences in SUD withdrawal. Future investigations focused on glial pathologies could uncover sex-specific, targetable mechanisms to improve treatment and promote sustained remission in both males and females.

## 1. Introduction

Substance Use Disorder (SUD) is an umbrella term used to describe conditions where an individual is unable to control use of a legal or illegal substance and progresses through stages of dependence, increased tolerance, and addiction (Van Etten & Anthony, 1999). Among the most prevalent and well-characterized SUDs are Alcohol Use Disorder (AUD), Cannabis Use Disorder (CUD), and Opioid Use Disorder (OUD). Each disorder involves substance-specific mechanisms and receptor pharmacology, but shares the core clinical features of drug initiation, escalation, withdrawal, and relapse.

Evidence for sex differences in disease and relapse vulnerability, symptom experience, and treatment response for SUDs is robust across both clinical and preclinical studies (Fonseca et al., 2021; Towers et al., 2023; Van Etten et al., 1999). Epidemiological studies show that males are two to three times more likely to initiate drug use and be diagnosed with SUD compared to females. In contrast, females progress more quickly through the stages of the disorder, report greater withdrawal severity, and are more likely to relapse compared to males. Incidence of SUD in females is also complicated by systemic barriers such as childcare responsibilities, stigma, and higher rates of co-occurring psychiatric illness, which make sustained remission more difficult (Barbosa-Leiker et al., 2021; Concato et al., 2024; Cooper & Craft, 2018; Peltier et al., 2019; Quality, 2017; White, 2020). Despite this epidemiological evidence for sex differences in SUDs, addiction research has been biased towards male-only cohorts, resulting in a limited understanding of sex-specific risk factors and etiologies.

Hypotheses for the pathology of SUDs have largely been neuron-centric, proposing roles of specific neuronal subtypes and receptors in addictive behaviors. However, over the past 20 years, glial cells (astrocytes, microglia, and oligodendrocytes) have emerged as equally important contributors to the neurobiological regulation of behavior, including the regulation and mediation of drug use (Rahman et al., 2022). While recent studies have begun to explore and emphasize the crucial role of glia in shaping reward- and substance use-related behaviors, sex differences in glial cell structure and function that may underlie drug use remain underexplored (Bachtell et al., 2017; Linker et al., 2019). Further, the largest sex differences observed in both clinical and preclinical SUD cohorts occur during withdrawal, yet most research on withdrawal has occurred in males. Sex differences in withdrawal include symptoms likely mediated by glial cells, including differences in immune response and autonomic nervous system (ANS) functioning. Thus, differences in glial cell structure and function are uniquely positioned as a mechanism to explain sex differences in substance use withdrawal. This review aims to attenuate this research gap by synthesizing the literature that posits glial cells as key mediators of sex differences. We will begin with an overview of the mechanisms underlying biological sex differences in the central nervous system (CNS) and in homeostatic glial cells, followed by a discussion of glial sex differences during withdrawal from substance use.

## 2. Baseline sex differences

### 2.1. Approaching sex differences in neurobiology

Sex differences in neurobiology arise due to a complex, bidirectional interaction between gonadal sex and chromosomal sex. Gonadal sex, referring to the presence of ovaries, testes, or some combination thereof, produces sex differences by influencing the production of sex-specific hormones. Chromosomal sex, referring to the presence of X and Y chromosomes, produces sex differences by influencing gene expression of X and Y-linked genes and by conferring epistatic interactions with genes located on autosomes (non-sex chromosomes). Differentiating the contributions of gonadal sex and chromosomal sex to canonical ‘male’ and ‘female’ sex differences is one of the most challenging frontiers in sex difference research of the present day.

Research in neurobiology has become increasingly interested in measuring sex differences after experimental manipulations. However, many experimenters fail to consider that their outcome variables (e.g., cell morphology, gene expression, behavior) likely start at a sex-specific baseline determined by hormonal and chromosomal biology. Measuring variables only after manipulations, without a baseline homeostatic comparison, may confound results and interpretations. The following sections outline the hormonal and chromosomal contributions to biological sex differences at homeostasis. These sex differences in receptor expression, hormone abundance, gene expression, and epistasis subsequently drive sex-specific behavioral vulnerabilities.

### 2.2. Hormonal sex differences

Hormones are the largest driver of sex differences in the central nervous system (CNS). While all hormones are present in both males and females, each hormone differs by sex in its abundance, timing, cycling, and site(s) of production (McEwen & Milner, 2017). These differences contribute to corresponding sex differences in hormone-regulated biological processes, including metabolism, growth and development, immunity, mood, behavior, and sleep-wake cycles (Hiller-Sturmhofel & Bartke, 1998). The three most important hormones that determine sex differences in the nervous system are estrogens, androgens, and progesterone (Hyer et al., 2018).

#### 2.2.1. Sex differences in CNS hormone receptor expression

Estrogens and estrogen-like molecules exert neuroprotective effects in the CNS by binding to estrogen receptors (ERs) ERα, ERβ, and G protein-coupled estrogen receptor 1 (GPER-1). Estrogen binding modulates cellular responses to oxidative stress, generally increasing the cell’s antioxidant activity (Almey et al., 2015). ER abundance and density vary greatly by receptor type, brain region, cell type, age, and sex (Lana et al., 2023). Female brains express larger numbers of ERs than male brains, but the specifics differ greatly by brain region. This higher expression of ERs in female brains is hypothesized as a key mechanism underlying sex differences in behavior (Goodman et al., 2023; Warfvinge et al., 2020).

Androgens also exert neuroprotective effects in the CNS. Binding to the singular androgen receptor (AR) induces similar antioxidant effects to estrogens, attenuating oxidative stress in cells (Duong et al., 2020). The abundance of ARs in the CNS also varies greatly by brain region, cell type, age, and sex. Generally, although AR expression shows less variability across regions than ER expression, males have higher AR expression than females in the hypothalamus and amygdala, but not in other brain areas (Fernandez-Guasti et al., 2000; Kruijver et al., 2001; Rybka et al., 2023). Differences in AR expression between males and females are hypothesized as another key mechanism underlying sex differences in behavior (Mhaouty-Kodja, 2018).

Progesterone is the third major class of hormones that determines sex differences in the CNS using neuroprotective mechanisms. Neuroprotection by progesterone is primarily accomplished through genomic mechanisms that upregulate neurotrophin expression, thus promoting cell survival. It is additionally known to act through its metabolites (e.g., allopregnanolone) using non-genomic mechanisms that activate cell signaling pathways and mediate neuroprotective effects (Singh & Su, 2013). Receptors for progesterone include classical nuclear progesterone receptors (PRs), as well as non-classical membrane progesterone receptors (mPRs) and splice variants such as progesterone receptor membrane component 1 (PGRMC1) (Petersen et al., 2013). Sex differences in PR expression are more nuanced than for ERs and ARs. PR expression can be regulated by ER expression or by androgen exposure, depending on the developmental time point (Quadros et al., 2002; Wagner et al., 2001). Brain region-specific PR expression has also been differentially observed in males and females (Yang et al., 2013).

#### 2.2.2. Sex differences in CNS hormone synthesis and abundance

Estrogen levels in the CNS are determined by an animal’s develop mental stage and by the menstrual/estrous phase in adult females. Prepubertal males and females both have very low levels of estrogen in the brain, with females showing higher levels than males in both rodents and humans (Bell, 2018; Courant et al., 2010; Cutler, 1997). At the onset of puberty, the ovaries begin producing large amounts of estrogen in females, which then fluctuates during the menstrual/estrous cycle throughout the life of the animal until menopause (Cioffi et al., 2024; Parker & Mahesh, 1976). Aside from the estrogens produced directly from the ovaries and indirectly from the testes that enter the brain through the Blood-Brain Barrier (BBB), both males and females produce brain-derived estrogens using the enzyme aromatase. Expression of aromatase varies by brain region, with females generally producing more brain-derived estrogens than males (Brann et al., 2022; Hu et al., 2023).

Androgens, primarily testosterone, are far more abundant in the brains of males compared to females. This is true in most (if not all) brain regions. The male dominance of CNS androgens is well established in both rodents and humans (Zuloaga et al., 2020). Sex differences in androgen abundance are primarily due to the large difference in gonadal production of testosterone between males and females. Differences in these circulating testosterone levels from the gonads drive differences in the number of androgens that cross the BBB.

Differences in progesterone levels between males and females vary depending on the phase of the menstrual or estrous cycle. In humans, progesterone levels during the menstruation and follicular phases in females are similar to homeostatic levels of progesterone in males, whereas during ovulation and the luteal phase, progesterone levels in females are vastly higher than in males (Singh & Su, 2013). Rodent estrous cycles are also correlated with differences in progesterone levels, but are more difficult to study due to the short nature of estrous phases. Temporal oscillations in the sexual dimorphism of progesterone activity require that the menstrual/estrous cycle be accounted for when measuring sexually dimorphic behaviors (Chari et al., 2020; Zeng et al., 2023).

### 2.3. Chromosomal sex differences

Sex chromosomes are also key drivers of CNS sex differences. Studies examining disorders of sexual development and sex chromosome aneuploidies have revealed differential influences of the X and Y chromosomes on brain development (Bakker, 2019; Green et al., 2019; Rahmadi et al., 2023). The presence of an X or Y chromosome has specific genetic effects, with certain genes being present only on the X chromosome or only on the Y chromosome. The number of X and Y chromosomes in an individual’s karyotype has downstream effects on the expression of gonadal hormones. Genetically engineered mouse models such as the Four Core Genotype (FCG) are currently used as tools for studying sex chromosome epistasis (De Vries et al., 2002).

### 2.4. Homeostatic sex differences in glia

**Fig. 1.**
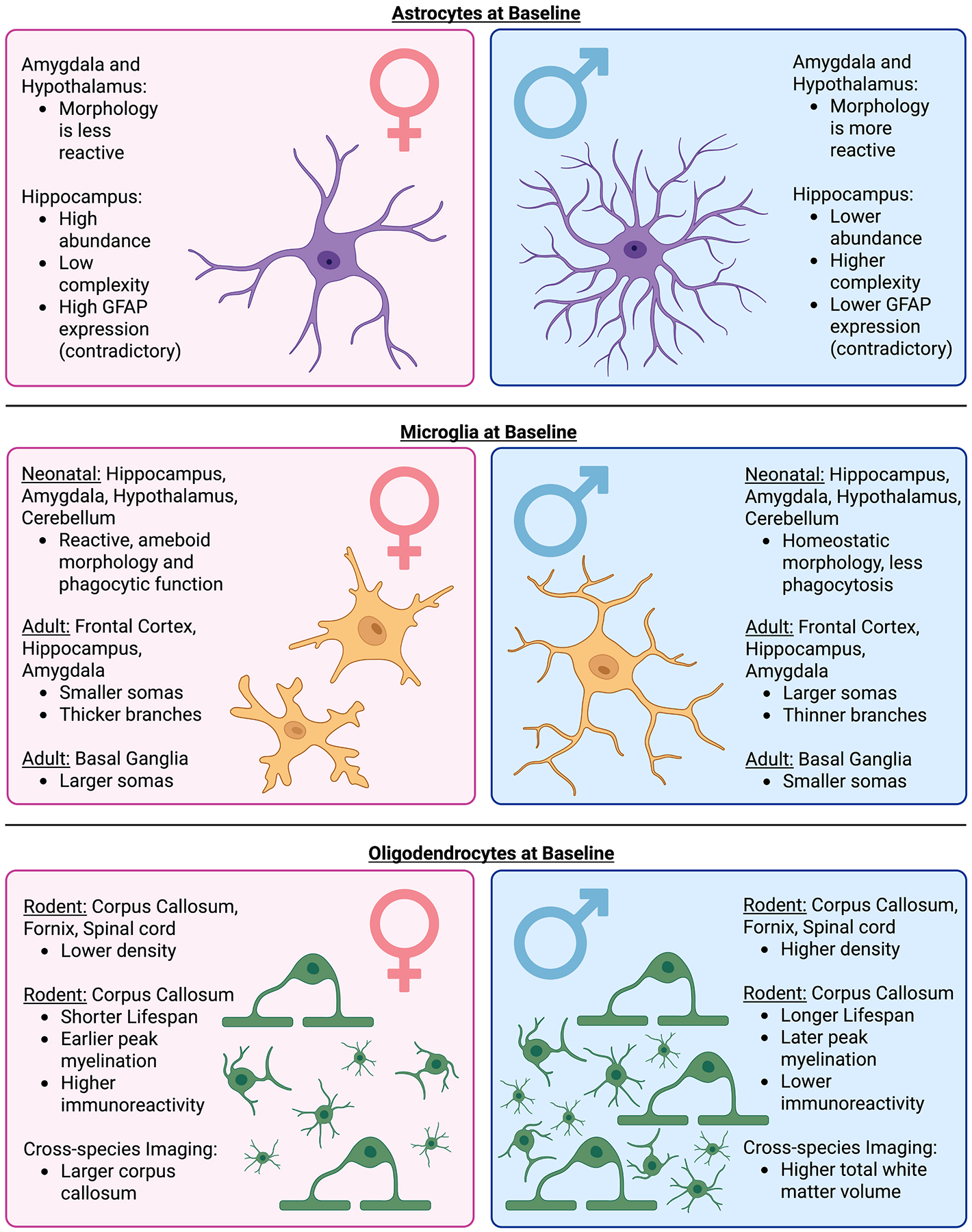
Sex-specific homeostatic baselines of glia structure and function. Astrocytes (Top), microglia (Middle), and oligodendrocytes (Bottom) exhibit sex-specific structure and function in homeostasis. Female data is summarized in the pink boxes. Male data is summarized in the blue boxes. Images of glial cells represent generalized, approximate morphology for each sex by cell type scenario. Created in BioRender. Keefauver,T.(2026) https://BioRender.com/04xsnmb.

*Astrocytes, microglia, and oligodendrocytes all exhibit baseline sex differences in structure and function*. Generally, glial cells in females exhibit more reactive, proinflammatory phenotypes at baseline, while glial cells in males exhibit more homeostatic phenotypes. However, measurements of sex differences in reactivity differ greatly based on age, brain region, and technique for measuring (i.e., gene expression vs. morphology). Key sex differences in glial structure and function at baseline are summarized in Fig. 1.

Astrocytes exhibit sex differences in their number, complexity, and receptor expression for estrogens, androgens, and progesterone, thus driving differences in function (Hidalgo-Lanussa et al., 2024; Johnson et al., 2008; Johnson et al., 2012). Astrocyte morphology is used as an index of functional reactivity, with more reactive astrocytes exhibiting increased complex branching (hypertrophy) and increased glial fibrillary acidic protein (GFAP) expression (Baldwin et al., 2024; Escartin et al., 2021; Gottipati et al., 2020; Lewis et al., 2008; Sofroniew, 2020; Verkhratsky et al., 2023). Morphological sex differences in astrocytes have been characterized in the hypothalamus, amygdala, and hippocampus (Gozlan et al., 2024; Schwarz & Bilbo, 2012). In the posterodorsal medial amygdala and hypothalamus, astrocytes from males are more abundant and more complex than females, indicating that males have a more reactive phenotype (Johnson et al., 2008; Mong et al., 1999; Mong & McCarthy, 2002). In the hippocampus, astrocytes from females are more abundant, less complex, and express higher levels of GFAP compared to males (Arias et al., 2009; Beyer et al., 1990; Mouton et al., 2002). This finding is difficult to interpret, since high astrocyte complexity is usually correlated with high GFAP expression. In this case, the astrocyte complexity and GFAP expression contradict one another and do not contribute to the same phenotypic characterization. This contradictory phenotype of astrocytes in the hippocampus warrants further investigation.

Sex differences in astrocyte morphology are thought to be functionally driven by estrogen-astrocyte interactions (Chisholm & Sohrabji, 2016; Fuente-Martin et al., 2013; Gillies & McArthur, 2010). Cultured astrocytes from female rodents show differences in reactivity to estradiol and perinatal testosterone, contributing to the increased neuroprotective capabilities of astrocytes in females (Acaz-Fonseca et al., 2016; Frago et al., 2017; Rurak et al., 2023; Santos-Galindo et al., 2011; Wu et al., 2016). Similarly, astrocytes from the developing mouse brain reach a mature state more quickly in males than in females (Rurak et al., 2022). A recent review by Vivi & Benedetto proposes that differences in astrocyte maturation timelines may drive sex differences in the prevalence of neurological disorders due to differences in critical period timing (Vivi & Di Benedetto, 2024). However, there is yet to be direct causal evidence for the role of baseline sex differences in astrocytes in neurological disorders (Bortolanza et al., 2025; Rurak et al., 2021).

Microglia exhibit a wide variety of sex differences unique to specific brain regions and developmental time points (Bishnoi & Bordt, 2025; Bobotis et al., 2023; Lyamtsev et al., 2025; Villa et al., 2018). Similar to astrocytes, microglia morphology is used as an index of functional reactivity. Homeostatic microglia are characterized by smaller cell bodies and numerous ramified processes. Reactive microglia exhibit a variety of proinflammatory and phagocytic functions and are characterized by larger cell bodies and few, short processes. Additionally, higher ionized calcium binding adaptor 1 (Iba1) abundance is associated with higher reactivity (Paolicelli et al., 2022). While there are morphological sex differences in rodent microglia in the frontal cortex, preoptic area, hippocampus, amygdala, hypothalamus, basal ganglia, and cerebellum at various developmental timepoints, not all developmental stages have been characterized for each brain region (Bishnoi & Bordt, 2025). The two best-characterized developmental stages are the neonatal period and adulthood, with neonatal microglia exhibiting the highest heterogeneity that greatly reduces at puberty (Han et al., 2021; Nelson & Lenz, 2017; Raju & Smith, 2025; Schwarz et al., 2012; Wade et al., 2017). In neonatal hippocampus, amygdala, hypothalamus, and cerebellum, microglia from females exhibit more amoeboid morphology with shorter branches and phagocytic tendencies compared to males (Lenz et al., 2013; Nelson et al., 2017; Perez-Pouchoulen et al., 2015; Schwarz et al., 2012; Weinhard et al., 2018). In the adult frontal cortex, hippocampus, and amygdala, microglia from females exhibit smaller soma size and thicker branches compared to males (Bollinger et al., 2016; Guneykaya et al., 2018; Schwarz et al., 2012). The opposite is true in the adult basal ganglia, where males exhibit smaller somas (Ali et al., 2024). Additionally, there is robust evidence that microglia in the cortex, hippocampus, and amygdala develop more quickly in male rodents compared to females (Schwarz et al., 2012), and that this difference makes males more susceptible to neonatal immune challenges (Bilbo et al., 2008; Bilbo et al., 2005; Bilbo et al., 2006; Lyamtsev et al., 2025; Raju & Smith, 2025; Williamson et al., 2011). Similar to the hypothesis by Vivi & Benedetto for astrocytes, these region- and development-specific differences in microglia morphology are hypothesized as a mechanism that may drive sex-specific susceptibilities to neurological disease due to differences in critical period timing (Schwarz & Bilbo, 2012).

Functionally, sex differences in microglia are hypothesized to be due to gonadal hormone signaling (Goodman et al., 2023; Villa et al., 2018), with increased estrogen exposure in females yielding a neuroprotective effect (Saeed et al., 2021; Vegeto et al., 2001; Vegeto et al., 2002; Villa et al., 2018; Villa et al., 2016; Zhang et al., 2012). However, increasing transcriptomic evidence suggests microglia maintain some sex-specific characteristics (e.g., gene expression) throughout the life of the cell, and that these characteristics remain unchanged even after transplantation of the microglia into the brain of the opposite sex (Barko et al., 2022; Guneykaya et al., 2018; Villa et al., 2018). Transcriptomic studies support the idea that chromosomal sex may be a key driver of sex differences in microglia structure and function. Alternate proposed mechanisms for mediating non-gonadal sex differences in microglia include sex differences in CNS gene expression for apoptotic programming and chemotactic signaling (Nelson & Lenz, 2017; Schwarz et al., 2012). Sex differences in microglia are likely driven by both gonadal hormones and chromosomal sex. Still, the relationships between gonadal hormones, genetics, and microglia are undoubtedly complex and poorly understood, highlighting the untangling of mechanisms underlying microglia sex differences throughout development as an important area of future research.

Baseline sex differences in oligodendrocytes and oligodendrocyte precursor cells (OPCs) are not well characterized (Akay et al., 2021; Yasuda et al., 2020). This is a limitation of studying oligodendrocytes in the context of neurological disorders, as observations of sex differences at baseline are necessary to interpret phenotypic sex differences after disease. The few studies that have examined baseline sex differences in oligodendrocytes and OPCs find similar patterns to microglia and astrocytes, where oligodendrocytes and OPCs express steroid hormone receptors and differentially respond to gonadal hormones (Cerghet et al., 2006; Marin-Husstege et al., 2004; Swamydas et al., 2009; Takao et al., 2004). Histological studies show that in the rodent corpus callosum, fornix, and spinal cord, oligodendrocyte density is greater in males than females, and these differences are maintained across developmental stages (Cerghet et al., 2006). Measures of proliferation and apoptosis suggest that the lifespan of oligodendrocytes is shorter in female rodent corpus callosum compared to males (Cerghet et al., 2006). Single-cell transcriptomics studies demonstrate that OPCs from female rodents express higher levels of proliferation and neuronal differentiation-associated genes, whereas OPCs from male rodents are enriched for markers related to cellular respiration (Beiter et al., 2022; Yasuda et al., 2020). This is in agreement with cross-species findings demonstrating that females have earlier peak myelination and higher immunoreactivity, indicating sex differences in the dynamics of OPC proliferation and differentiation (Benes et al., 1994; Yurgelun-Todd et al., 2002). Additional cross-species imaging characterizes a higher total white matter volume in males, but a larger corpus callosum area in females (Gur et al., 1999; Holloway & de Lacoste, 1986). The functional implications of these identified structural differences, transcripts, and pathways remain to be studied.

## 3. Sex differences in withdrawal symptoms

### 3.1. Somatic vs. affective withdrawal

Symptoms of SUD withdrawal can be divided into two categories: somatic withdrawal, which includes the physical symptoms of a drug-free period, and affective withdrawal, which denotes the emotional and mental symptoms of the withdrawal phase. Although both types of symptoms frequently coexist within SUD populations, the prevalence of these two domains differs by sex, by temporal phase of withdrawal, and by substance, including alcohol (Canver et al., 2025; Kattimani & Bharadwaj, 2013), cannabis (Connor et al., 2022), and opioids (Koob et al., 1992; Shah & Huecker, 2025).

**Fig. 2.**
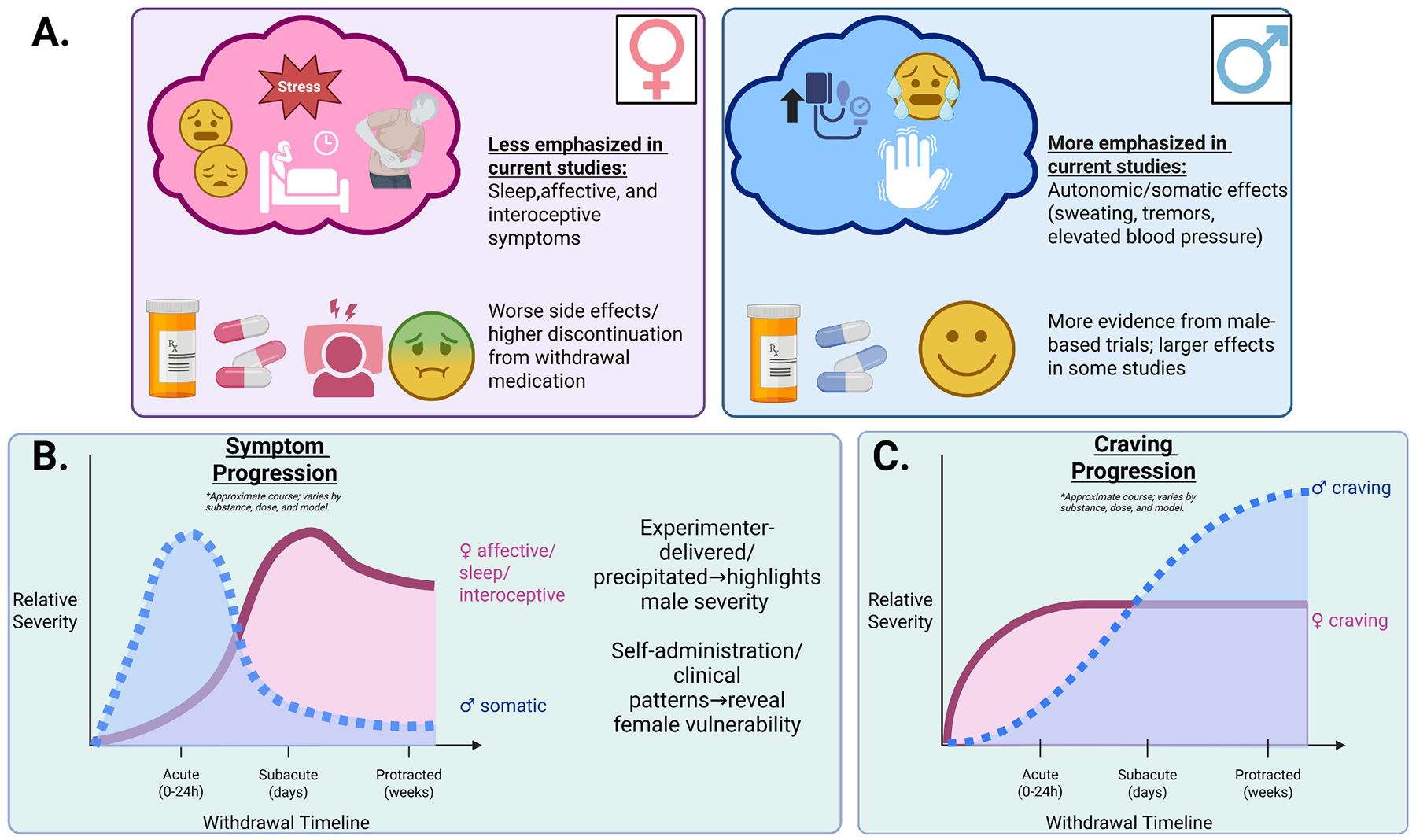
Clinical sex differences in withdrawal symptoms and time course. **(A)** Differences in symptoms exhibited by females and males, including effects of withdrawal medication. **(B)** Approximate differences in symptom progression between females and males. Female portion shows approximate progression of affective/sleep symptoms during SUD. Male portion shows approximate progression of somatic symptoms during SUD. **(C)** Approximate differences in craving progression between females and males in SUD. Pink = female. Blue = Male. Created in BioRender. Horan, N. (2026) https://BioRender.com/3jl4p23.

In clinical settings, females often report greater overall burden of withdrawal, driven more frequently by affective and interoceptive symptoms (e.g., gastrointestinal distress, negative affect, and sleep issues) compared to males (Herrmann et al., 2015; Unlu et al., 2023; van Wijk & Kolk, 1997; Weinberger et al., 2016). Furthermore, these sex differences are temporally regulated: *males are more likely to exhibit heightened somatic signs during precipitated or early withdrawal, whereas females are more likely to exhibit heightened affective symptoms during spontaneous or prolonged withdrawal* (Bobzean et al., 2019; He et al., 2020; Jones et al., 2022; Kokane and Perrotti, 2020; Unlu et al., 2023). While some clinical studies find no consistent differences in the duration, intensity, or length of withdrawal between males and females when looking at AUD (O’Connor et al., 1993; Unlu et al., 2023), or in mixed studies including methamphetamines and heroin (He et al., 2020), other reports indicate that females experience greater withdrawal severity or require longer and more resource-intensive hospitalizations for alcohol (Canales et al., 2022; Unlu et al., 2023) and opioids (Ware et al., 2022). Sex differences in withdrawal presentation carry critical clinical implications. Both somatic and affective symptoms introduce barriers to remission, with people returning to drug use to alleviate distressful physical, emotional, and mental withdrawal symptoms. Still, withdrawal treatments and management strategies emphasize somatic symptoms (tremors, sweating, blood pressure, seizures) and therefore are more efficacious in males. Affective and interoceptive symptoms (e.g., sleep disturbances, gastrointestinal distress, and mood disturbances), which are more prevalent in females, remain under-recognized and under-treated in SUDs (Fig. 2A). If withdrawal in females is more cyclic, hormone-sensitive, and affectively intense, then a “one-size-fits-all” approach may be unsuitable to address symptoms in both sexes. Poorly managed withdrawal is known to increase the risk of relapse, but current treatment models fail to address the sex-specific symptoms. This is especially important for females who return to substance use not only to avoid physical discomfort, but also to escape the affective distress they are more likely to experience.

Sex differences in withdrawal severity are further compounded by pharmacologic disparities. Most pharmacotherapies and withdrawal treatments have been developed and validated using male-dominant cohorts. Accordingly, drugs may have unforeseen side effects or diminishing efficacy in females, making adherence to therapeutic schedules and sustained remission more difficult (McRae-Clark et al., 2016; McRae-Clark et al., 2015). For example, naltrexone for AUD is equally effective for females and males; however, females report greater adverse events, including nausea and sleep disturbances, which together represent crucial barriers to adherence to treatment (Baros et al., 2008; Suh et al., 2008). Not all medications show a male bias; some, such as baclofen, have better outcomes in females with AUD than in males (McKee and McRae-Clark, 2022; Morley et al., 2022). Similar patterns have been observed with other pharmacologic agents, where efficacy often diverges by sex or hormonal state, suggesting that neurobiological and endocrine factors jointly shape treatment responsiveness. Yet, many studies still fail to analyze or report sex-stratified outcomes, limiting clinical insight and the potential for precision treatment.

These inconsistencies highlight the need for translational models that better capture both female vulnerability to SUDs and affective symptoms. However, translating the findings and symptomology from clinical work to preclinical studies is complex. Results from preclinical studies examining potential sex differences depend heavily on experimental design. In experimenter-delivered or naloxone-precipitated withdrawal models, a dose of the opioid antagonist naloxone is rapidly administered to morphine-dependent mice, causing the sudden and intense expression of physical withdrawal symptoms like jumping, urination, and diarrhea. In this model of withdrawal, male rodents often display more robust somatic signs than females (Bobzean et al., 2019; Craft et al., 1999; Tan et al., 2019). However, these experimenter-administered models may underestimate withdrawal severity in females, as self-administration models, which more closely resemble human drug-taking patterns and withdrawal, have revealed greater withdrawal severity in female rodents compared to males. For example, female rats that self-administer fentanyl exhibit enhanced somatic signs of OUD withdrawal compared to males at both acute and protracted timepoints (Fig. 2B) (Towers et al., 2022b; Townsend et al., 2021). Patterns of craving also vary by sex, with female rodents exhibiting earlier onset of craving that flattens, while males exhibit a gradual escalation of craving over time (Towers et al., 2022a) (Fig. 2C).

Clinical and preclinical findings converge on a key insight: withdrawal mechanisms, symptoms, and treatment responses are not uniform across sexes. *Incorporating sex as a biological variable in both clinical and preclinical research is critical to developing precision treatments that improve long-term remission outcomes for all individuals*.

## 4. Global contributions of glia to withdrawal

### 4.1. Sex differences in global glial responses to withdrawal

**Fig. 3.**
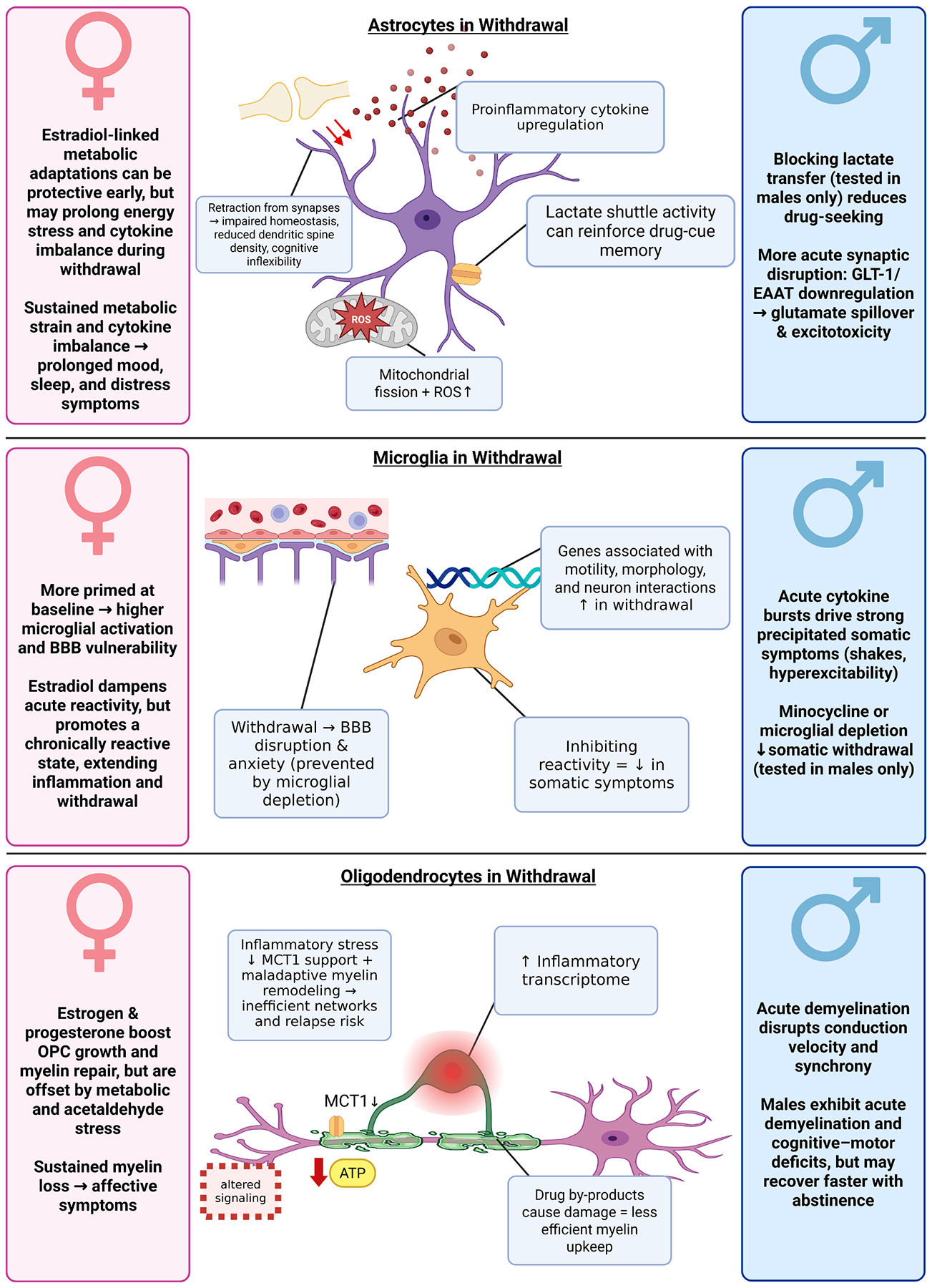
Sex-specific changes in glia structure and function during withdrawal. Astrocytes, microglia, and oligodendrocytes respond differently in males and females to withdrawal. **(Top)** In females, estradiol-linked metabolism provides early protection for astrocytes but extends stress and inflammation. This results in mitochondrial strain, production of reactive oxygen species (ROS), synaptic loss, and ongoing symptoms. Males show larger glutamate spillover and excitotoxicity. Blocking lactate transfer decreases drug-seeking behavior. **(Middle)** Microglia from females remain chronically reactive, which produces blood-brain barrier (BBB) disruption and anxiety. In contrast, male microglia are associated with sudden spikes in cytokines and somatic symptoms. **(Bottom)** Oligodendrocyte stress reduces MCT1 support and myelin efficiency: females show sustained myelin loss and affective symptoms, whereas males display acute demyelination with faster recovery. Created in BioRender. Horan, N. (2026) https://BioRender.com/jp7xn8y.

While previous research on SUDs has focused on neurons as the center of addictive-like behavior, more studies are shifting focus to glia. Glial cells’ ability to fine-tune neurons and control the cellular environment could mediate the observed sex differences in complex craving and withdrawal. Notably, a clinical trial of ibudilast, a glial cell modulator that attenuates microglial production of IL-6 and TNF-α and reduces astrocyte reactivity, reported decreases in subjective ratings of opioid withdrawal symptoms (Cooper et al., 2016). However, this study was almost exclusively conducted in males (26 M: 4F). *Since glia are known to respond differently across sexes and hormonal states, glial mechanisms may explain why females and males experience withdrawal differently*. Fig. 3 illustrates these sex-specific adaptations, summarizing astrocyte, microglia, and oligodendrocyte contributions to withdrawal. Because females often exhibit greater withdrawal-related affective symptoms and poorer treatment response, understanding glial contributions may be particularly important for improving outcomes in females. *Investigating withdrawal through a glial lens could uncover sex-specific, targetable mechanisms to improve treatment and promote sustained remission in females*.

### 4.2. Astrocyte contributions to withdrawal

Across rodent models and cell culture studies, astrocytes emerge as key regulators of glutamate clearance and metabolic support. These functions become dysregulated during withdrawal in ways that destabilize synapses and promote relapse across different sub-categories of SUD (Rose & Chatton, 2016; Scofield & Kalivas, 2014). During withdrawal, astrocytes exhibit several maladaptive alterations, including downregulation of EAAT2/GLT-1 and retraction of processes from synapses in both OUD (Kruyer & Kalivas, 2021) and general SUD (Takahashi et al., 2015), although these reviews highlighted mostly male findings or did not report sex differences. Resulting glutamate spillover and excess activation of extrasynaptic NMDA receptors impairs plasticity and dendritic stability, producing clinical correlates such as anxiety, irritability, and seizure vulnerability (Ayers-Ringler et al., 2016; Gutierrez & Staehle, 2015). In addition to excitotoxic signaling, withdrawal heightens astrocytic metabolic strain, characterized by mitochondrial dysfunction, increased reactive oxygen species (ROS), and upregulation of pro-inflammatory cytokines that further potentiate neuronal hyperexcitability (Kumar et al., 2024b; Singh et al., 2025). Additionally, under normal conditions, astrocytes supply lactate to neurons for plasticity and memory consolidation (Suzuki et al., 2011). During withdrawal from cocaine, this same “lactate shuttle” helps consolidate drug-associated memories and cues (Boury-Jamot et al., 2016). Blocking lactate transfer selectively reduces the strengthening of drug-related circuitry and curbs drug-seeking behavior, although this has only been demonstrated in males (Chen et al., 2023). Primary astrocyte cultures from female rodents demonstrate that sex hormone exposure significantly alters astrocytic structure and function. Estradiol promotes astrocyte proliferation and enhances resilience to cellular stressors such as oxidative damage, lipotoxicity, and inflammation (Santos-Galindo et al., 2011). Estradiol also enhances mitochondrial efficiency and glycolytic capacity, allowing astrocytes to sustain neuronal energy demands. Estradiol has further been shown to increase astrocytic GLT-1 and AQP4 expression, thereby supporting glutamate clearance and water balance (McInvale et al., 2025). These functions could mitigate excitotoxicity and osmotic stress during withdrawal in females. Contrary studies indicate that acute estradiol signaling through ERα can reduce glutamate uptake, which may amplify glutamate spillover and drug cue reactivity (Sato et al., 2003). Such divergent findings highlight the context-dependent influence of estradiol on astrocytes, with potential for both protective and maladaptive effects during different phases of withdrawal (Itoh et al., 2023).

Testosterone also shapes astrocytic physiology, increasing AQP4 expression and reducing pro-inflammatory secretion (Gu et al., 2003; Turniak-Kusy et al., 2024). However, direct effects of testosterone on astrocytic glutamate transport remain largely untested, and most available evidence comes from studies using anabolic-androgenic steroids. Supraphysiological androgen exposure, with anabolic steroids like nandrolone, has been associated with reduced GLT-1 expression and impaired glutamate clearance, suggesting that excessive androgenic activity may exacerbate glutamate spillover and relapse vulnerability (Kalinine et al., 2014; Rodolphi et al., 2021). Together, these findings suggest that estradiol may buffer against long-term excitotoxicity while contributing to metabolic vulnerability and protracted withdrawal, whereas testosterone may exacerbate acute hyperexcitability but allow faster recovery. *Such hormone-dependent mechanisms may underlie observed clinical sex differences in withdrawal, with males exhibiting stronger precipitated symptoms and females reporting more persistent sleep and mood disturbances*.

### 4.3. Microglia contributions to withdrawal

Prior reviews have thoroughly summarized the general roles of microglia in substance use withdrawal (Bachtell et al., 2017; Bergkamp & Neumaier, 2025), but neither provided extensive mechanistic coverage of sex-specific glial pathways. Clinical and preclinical studies converge to implicate microglia in the inflammatory symptoms of withdrawal. In clinical populations, elevated pro-inflammatory cytokines correlate with craving at the onset of alcohol detoxification and normalize with treatment (Leclercq et al., 2012). Postmortem human data further indicate that microglia in females are more “reactive” than in males (Yoblinski et al., 2025), suggesting that sex-specific differences in baseline microglial reactivity could influence withdrawal trajectories.

Preclinical rodent models give further insight into sex-specific roles of microglia in withdrawal. Withdrawal, specifically within OUD, is characterized by upregulation of genes associated with microglial motility, morphology, and neuron-microglia interactions (Coffey et al., 2022). These changes heighten neuroimmune signaling and drive neural hyperexcitability that manifests as somatic and affective withdrawal symptoms. Interestingly, preclinical studies indicate that estradiol can paradoxically dampen microglia activation in response to immune challenge (Wu et al., 2016). This combination may create a chronically primed and hormonally-modulated state that leaves females more vulnerable to protracted withdrawal. Microglia also shape withdrawal intensity through their interactions with the blood-brain barrier. Female mice exhibit higher baseline BBB permeability in the prefrontal cortex and hippocampus compared to males, and this difference is exacerbated during withdrawal from nicotine (Kumar et al., 2024a). Immune challenge increases microglial reactivity and compromises BBB integrity (Banks et al., 2015; Haruwaka et al., 2019), whereas microglial depletion prevents BBB disruption and attenuates withdrawal-induced anxiety from nicotine (Kumar et al., 2024a). Additionally, microglial depletion using a colony-stimulating factor 1 receptor (CSF1R) antagonist, PLX5622, reduces expression of proinflammatory genes such as TNF-α and Ccl2 and enhances expression of anti-inflammatory genes such as IL-1ra and IL-4 during ethanol withdrawal (Walter & Crews, 2017). Interestingly, males exhibit stronger acute inflammatory responses to immune stimulation, showing higher cytokine expression following lipopolysaccharide (LPS) exposure (Loram et al., 2012; Yanguas-Casas et al., 2018), consistent with the idea that acute cytokine surges may drive acute somatic symptoms seen in males. Increases in cytokine production are also linked to withdrawal symptoms: minocycline-mediated inhibition of microglial activity decreases somatic opioid withdrawal symptoms (Liu et al., 2020), and depletion of microglia reduces alcohol intake in dependent mice with no effect on nondependent mice (Warden et al., 2020). However, these findings are limited by their male-only cohorts.

Together, both clinical and preclinical findings suggest that females enter withdrawal with more globally primed microglia and greater BBB vulnerability, leading to prolonged, inflammation-driven trajectories. In contrast, males experience stronger acute cytokine surges, resulting in more intense precipitated somatic symptoms. *Microglia therefore, represent a keystone mechanism for divergence in withdrawal trajectories with therapeutic implications: interventions that blunt acute cytokine surges may be more efficacious in males, while interventions that dampen protracted BBB-driven inflammation may be more efficacious in females*.

### 4.4. Oligodendrocyte contributions to withdrawal

Prior reviews have summarized the broad roles of oligodendrocytes in addiction and withdrawal in both general SUD (Saba, 2023) and AUD (Ponomarev, 2013), showing that withdrawal induces an inflammatory oligodendrocyte transcriptome that disrupts white-matter homeostasis and axon-glial metabolic support. However, few studies have commented on sex-specific differences. Neuroimaging has consistently revealed abnormalities in white matter structures that remain present even after abstinence: Diffusion tensor imaging (DTI) reveals that fractional anisotropy is lower in brains of individuals suffering from alcohol or opioid dependence, and this decrease is associated with executive-function deficits, depressive symptoms, and relapse risk (Bava et al., 2009; Monnig et al., 2013; Yuan et al., 2009). While there are still very few sex-specific clinical studies, a few lines of emerging research indicate that females with AUD have a more pronounced loss of myelin, and their white matter is slower to recover in the early period of abstinence (Alhassoon et al., 2012; Pfefferbaum et al., 2015). These data emphasize disruption of myelin as an understudied component of withdrawal and remission. Importantly, functional consequences of myelin loss during withdrawal may depend on sex-specific baseline differences in white matter organization. Males and females differ in baseline myelination patterns, axon caliber, and gray-to-white matter ratios across multiple brain regions, including the corpus callosum and frontal cortex. As a result, equivalent degrees of demyelination during withdrawal may not have equivalent consequences in males and females, complicating direct comparisons of magnitude alone.

Preclinical work supports and extends these observations. Demyelination is correlated with impaired cognition and motor performance in rodents (Huang et al., 2024a), although this evidence is from limited studies of males in nicotine withdrawal. Some demyelination may recover with abstinence or pharmacologic intervention. However, this study highlights the vulnerability of myelin during withdrawal and the behavioral consequences of disrupted white matter function. Preclinical models have also demonstrated that sex and hormones strongly affect myelination patterns in withdrawal. Ethanol is broken down by the body into acetaldehyde, a toxic byproduct that damages oligodendrocytes and disrupts the upkeep of myelin (Coutts & Harrison, 2015). Several lines of research suggest that sex and hormones shape vulnerability to acetaldehyde-related myelin degeneration. Females may accumulate more acetaldehyde and experience greater oxidative stress than males (Frezza et al., 1990; Penaloza et al., 2020). Simultaneously, gonadal hormones play an important protective role. Both estrogen and progesterone affect OPC proliferation, myelin remodeling, and oligodendrocyte gene expression (Long et al., 2021), and progesterone supports oligodendrocyte survival and maintains myelin integrity under stress (El-Etr et al., 2015). This dichotomy of females experiencing greater metabolic stress but also stronger hormonal protection by estrogen and progesterone may help explain why alcohol studies report sex- and region-specific effects on myelinated axons (Rice et al., 2019; Silva-Gotay et al., 2021; Tavares et al., 2019), and why demyelination models frequently find females remyelinate more efficiently (Bardy-Lagarde et al., 2025). Likely, these findings can be explained by a dynamic balance, in which females’ heightened vulnerability to acetaldehyde is offset by hormones that support repair, while males are less susceptible to metabolic stress but exhibit lower levels of estrogen and progesterone hormone-driven resilience. Accordingly, sex differences in demyelination during withdrawal should be interpreted relative to baseline myelination and circuit organization, as similar changes may carry different functional consequences in males and females.

*The above findings highlight oligodendrocyte vulnerability as a probable contributor to key pathologies associated with withdrawal. Still, focused investigations targeting the sex-specific dynamics of oligodendrocytes are lacking*. Further investigations into all drug types are needed, as the majority of studies suggesting sex differences in myelination focus on alcohol.

## 5. Region-specific contributions of glia to withdrawal

### 5.1. Sex differences in region-specific responses to withdrawal

**Fig. 4.**
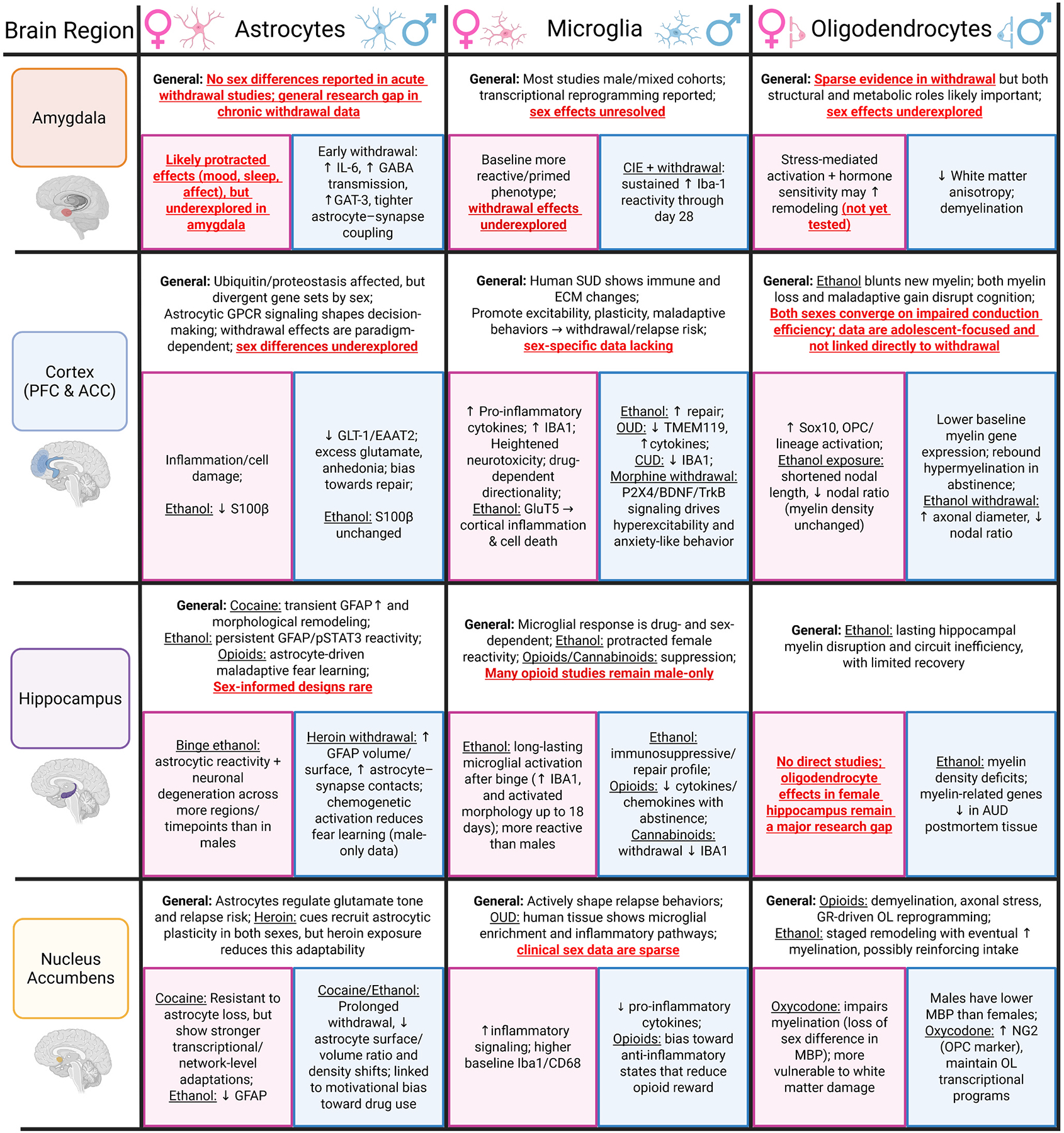
Summary of region- and sex-specific differences in glia during withdrawal. Sex differences in astrocytes, microglia, and oligodendrocytes are described across the amygdala, cortex, hippocampus, and nucleus accumbens. Non-sex-specific results are summarized in the white, general boxes. Observations unique to females are detailed in the pink boxes. Observations unique to males are detailed in the blue boxes. Drug subtypes are underlined. Gaps in current knowledge and underexplored areas are listed in red text throughout. Created in BioRender. Horan, N. (2026) https://BioRender.com/4zck5yz.

The amygdala, cortex (prefrontal cortex (PFC) and anterior cingulate cortex (ACC), hippocampus, and nucleus accumbens (NAc) are central nodes of the reward system. In both clinical and preclinical studies, these subregions show unique structural and functional changes during withdrawal. Some changes differ between males and females, pointing to sex-specific withdrawal-induced circuit changes in general SUD (Fang et al., 2022), AUD (Flook et al., 2021; Sawyer et al., 2017), and OUD (Kalamarides et al., 2023; Santoro et al., 2017). However, glial contributions within these distinct brain regions are understudied. Glial contributions are likely to be heterogenous, with different brain regions exhibiting unique alterations. Dissecting and synthesizing previous literature by brain region provides a clearer view of how sex and hormones influence glial responses. Fig. 4 summarizes sex-dependent, region-specific adaptations, highlighting how glial mechanisms vary across the amygdala, cortex, hippocampus, and NAc. *Here, we highlight how sex-specific, region-specific glial contributions drives sex-specific phenotypes in withdrawal*, focusing on astrocytic regulation of glutamate and cell support, microglial cytokine signaling, and oligodendrocyte-mediated conduction timing.

### 5.2. Amygdala-mediated withdrawal

*Due to its sensitivity to emotional and associative processing, the amygdala plays a key role in reward-seeking and relapse during withdrawal from both OUD* (Guo et al., 2024) *and general drug use* (Guo et al., 2024; Luo et al., 2013; Simic et al., 2021). The amygdala has clearly established sex differences: in humans, females exhibit prolonged activation of the amygdala in response to negative stimuli, indicating a bias that may increase stress and cue reactivity (Andreano et al., 2014). In studies of cocaine (Warlow et al., 2017) and opioid use (Wassum et al., 2016), this prolonged activation during withdrawal increases the perceived worth of drugs and can lead to maladaptive cue- or activation-induced seeking even in the absence of direct drug availability. This effect in females is further supported across substances by withdrawal-related circuit disinhibition in opioid (Gregoriou et al., 2023) and alcohol (Roland et al., 2023) models. Longitudinally, preclinical c-Fos studies in alcohol dependence show similar central amygdala (CeA) and basolateral amygdala (BLA) activity between sexes at 24 h of withdrawal; however, by 28 days, CeA activity decreases in both sexes, while BLA activation only persists in females (Li et al., 2019). Despite females exhibiting this prolonged amygdala activation that can lead to increased reward seeking, hormonal modulation appears partly protective. Targeted shRNA knockdown of estrogen receptor subtypes in the amygdala of female rats indicates that silencing ERβ increases anxiety-like responses to aversive stimuli (Le Moene et al., 2019), suggesting that ERβ signaling in the amygdala normally exerts an anxiolytic or stress-buffering effect. While this does not directly address drug withdrawal, it supports a role for estrogen in modulating amygdala circuits. This sex difference is especially relevant to the anxiety and mood-related symptoms seen in female withdrawal: if estradiol modulates the amygdala within anxiety-driven states, it may explain how prolonged amygdala activation during withdrawal can stimulate the more affective symptoms seen in females. The stronger amygdala activation observed in human females during alcohol withdrawal (Grodin et al., 2024) may reflect a scenario in which stress, withdrawal, or repeated cue reactivity overcomes or saturates the protective capacity of ERβ pathways. In contrast, males exhibit reduced total amygdala volume with alcohol dependence, a structural change not seen in females (Grace et al., 2021).

#### 5.2.1. Astrocytes in amygdala-mediated withdrawal

Current knowledge about astrocytes in amygdala-mediated withdrawal is derived from preclinical studies, as human postmortem research and clinical studies have not yet examined this topic. Preclinical research has implicated amygdala astrocytes primarily within neuroimmune signaling and glutamate regulation pathways. Astrocytes in the amygdala exhibit increased neuroinflammatory signaling after opioid withdrawal (O’Sullivan et al., 2019). During alcohol withdrawal, alterations in gamma-aminobutyric acid (GABAergic) signaling and elevated neuronal excitability are directly associated with astrocyte-derived IL-6 (Roberts et al., 2019). In addition to interfering with GABAergic signaling, this same study showed that a general increase in IL-6 is linked to a decrease in exploration of novel environments as well as an increase in depressive-like behaviors (Roberts et al., 2019). Additional adaptations during alcohol withdrawal include tighter astrocyte-synapse coupling and overexpression of the astrocytic GABA transporter GAT-3 (Nentwig et al., 2025). Overall, these astrocytic adaptations during withdrawal may represent a shared mechanism between substances that mediate neuronal excitability, converting inhibition signaling from a protective brake into a driver of pathological hyperactivity. However, most research to date has been performed in male or mixed-sex cohorts during acute (~24 h) withdrawal, when somatic symptoms and cytokine surges predominate. Studies extending into protracted withdrawal (up to 28 days) are limited, even though this window is critical for understanding female-specific affective and sleep-related outcomes (Bobzean et al., 2019). The few studies that have looked at hormonal effects have found interesting results. GABA’s anxiolytic effects are known to be hormone dependent. Fluctuations across the estrous cycle alter sensitivity to stress and to GABA-enhancing drugs such as benzodiazepines, suggesting that astrocytic adaptations affecting GABA signaling may have divergent consequences in males and females (Maguire & Mody, 2009; Pestana & Graham, 2023). However, because most existing work relies on male or mixed-sex cohorts, the influence of hormonal state on astrocytic regulation of amygdalar inhibitory tone remains largely unresolved.

Astrocytes also control the amygdala's excitatory drive by regulating glutamate. Silencing astrocyte activity attenuates ethanol-induced pre-synaptic glutamatergic activity, and activating astrocytes using LPS without ethanol stimulates glutamate transmission (Melkumyan et al., 2022). This suggests that astrocytes directly mediate ethanol’s influence on circuit function, providing evidence for astrocytes’ ability to regulate excitatory drive in the amygdala. However, this study was only completed in males, limiting the understanding of sex-specific glutamate dynamics.

Beyond their acute effects on excitatory and inhibitory signaling, amygdala astrocytes also appear to have modulatory influences on craving and drug seeking. Disrupting the transfer of lactate from astrocyte to neuron in the CeA diminishes cocaine craving after a period of withdrawal (Chen et al., 2023). Astrocyte lactate transfer in the BLA is also implicated in the reconsolidation of cocaine-conditioned place preference (Boury-Jamot et al., 2016), suggesting that astrocytes help maintain drug-related memories. However, it is unclear whether these lactate-shuttling mechanisms differ by sex. Changes in astrocytic GABA signaling, particularly involving GAT-3 in the central amygdala, occur following ethanol exposure but do not appear to contribute to escalated drinking behavior in rodents (Nentwig et al., 2025).

*Astrocytic circuits in the amygdala control craving, withdrawal, and relapse; however, it is unclear if there is sex-specificity*. Do hormones and/or withdrawal trajectories produce substantial differences in astrocyte responses to SUD that may explain the differences in affective symptoms during withdrawal? While astrocyte-induced changes in GABA, glutamate, and lactate shuttling may not be a “precursor” to relapse, they may still intensify affective symptoms. As we work to understand the role of astrocytes in withdrawal-like states, future studies should approach these questions from a sex-and trajectory-specific perspective.

#### 5.2.2. Microglia in amygdala-mediated withdrawal

Microglia are the brain's main immune sentinels, working in tandem with astrocytes to influence inflammation in a sex-specific manner. Most conclusions about amygdalar mechanisms of inflammation come from preclinical work, as human postmortem studies have yet to examine microglial changes in the amygdala during withdrawal. Studies investigating withdrawal from opioids and chronic intermittent ethanol vapor (CIE) show that microglia in the amygdala decrease expression of antioxidant genes, increase expression of pro-inflammatory Tnf-α, and increase expression of Iba-1 for up to 28 days (O’Sullivan et al., 2019; Sanchez-Alavez et al., 2019). However, in a drinking-in-the-dark model, amygdala microglia show no change in reactivity measured via Iba-1 immunoreactivity (Nelson et al., 2021). At the molecular level, microglia engage neuroimmune and cytokine programs during opioid dependence that are only partly reversed in withdrawal. 45% of the genes differentially expressed during dependence return to baseline, but these account for only ~30% of all genes altered during withdrawal, suggesting that withdrawal involves not only a reversal of dependence-related effects, but also substantial new transcriptional changes (Yan et al., 2023). All four studies investigated only male rodents but suggest that *microglial activation in the amygdala may be paradigm-dependent, with distinct transcriptional and functional trajectories across dependence and withdrawal*.

While most studies of withdrawal-induced microglial changes have been conducted only in males, emerging evidence suggests a potential sex-by-time interaction in which microglial activation states evolve differently across withdrawal in males and females. Microglia in female amygdala are less ramified at baseline, consistent with a more reactive phenotype that may affect responses to withdrawal and stress (Soares et al., 2025). When examining how this baseline sex difference drives SUD-related changes, one study found that after 15 days of a drinking-in-the-dark paradigm, amygdala microglial density and reactivity showed distinct sex- and stress-dependent patterns. In the BLA, ethanol reduces microglial density and CD68 expression. Stress reverses this ethanol-induced decrease in females, but in males, this reduction was only seen when ethanol exposure was paired with stress (Soares et al., 2025). This stress contingency may help explain why Nelson et al. (2021) did not observe microglial changes in drinking-in-the-dark paradigms. These findings indicate that ethanol exposure dampens microglial phagocytic activity, though the direction and magnitude of this effect depend on sex and stress exposure. Further, after an additional week of drinking in the dark, these effects are no longer present. This additional week of ethanol exposure increased amygdala microglial density in stressed males, highlighting dynamic and time-dependent regulation of microglial states across withdrawal. Reversal of the initial withdrawal-induced microglial reduction in males may be a key feature of amygdala sex differences.

#### 5.2.3. Oligodendrocytes in amygdala-mediated withdrawal

Emerging evidence supports a role for oligodendrocytes in the amygdala during withdrawal. Proper control of emotions, stress, and drug craving requires synchronized communication between the amygdala and other limbic regions. Human studies of opioid addiction show reduced anisotropy in amygdala-associated white matter tracts observed by DTI (Upadhyay et al., 2010), indicating decreased tract connectivity and potential demyelination. Oscillatory synchrony and timing across these circuits may become unstable due to conduction delays caused by myelin loss, intensifying withdrawal-related negative affect.

Rodent models further implicate amygdala oligodendrocytes in withdrawal. Transcriptomic profiling of the amygdala following voluntary ethanol drinking revealed enrichment of oligodendrocyte- and myelin-related pathways, including histone deacetylation-linked epigenetic regulation, and identified the oligodendrocyte-lineage transcription factor Sox17 as a hub regulator (Narendra et al., 2022). Viral vector-mediated knockdown of Sox17 in the amygdala prevented escalation of ethanol intake during repeated access, directly linking an oligodendrocyte-related gene to ethanol consumption in female mice (Narendra et al., 2022). Chronic psychological stress has also been shown to produce amygdala demyelination accompanied by anxiety- and depression-like behaviors, which can be partially rescued by systemic remyelination-promoting or anti-inflammatory treatments (Li et al., 2025). Given that chronic stress is both a precipitating factor and a consequence of substance use and withdrawal, these findings suggest that *stress-induced white matter disruptions in the amygdala may contribute to the affective disturbances that promote continued drug seeking or relapse*. Although these myelination pathologies were characterized within the amygdala, most interventions were systemic, which highlights the possibility that results were not amygdala specific. Sex-specific analyses are lacking, but a higher prevalence of stress-induced demyelination in females may explain the higher prevalence of affective symptoms and mood comorbidities during withdrawal.

#### 5.2.4. Glial interactions in amygdala-mediated withdrawal

Combined findings suggest that astrocytes, microglia, and oligodendrocytes all contribute individually to observed sex differences in amygdala activity during withdrawal. Evidence also suggests that interactions between glia mediate behavior. Astrocytes modulate excitability and synaptic tone in the amygdala via the release of cytokines and uptake of neurotransmitters, which may either amplify or buffer neuronal firing, depending on withdrawal phase and hormonal exposure. Microglia exhibit sex differences in their baseline morphology and activation thresholds; their responses to alcohol and opioids appear to be relatively prolonged in females, positioning microglia as an important mechanism for sustaining local inflammatory tone and the consequent regulation of astrocytic behavior through shared microglia-astrocyte cytokine pathways*. Microglia*–*astrocyte interactions, therefore, may prolong amygdala hyperresponsivity or reduce the resolution of early stress signals*. Oligodendrocytes, although less studied, play a critical role in maintaining the timing and synchrony of amygdala-containing circuits. Loss of myelin or metabolic deficits among females, especially during inflammation, may desynchronize these pathways, impairing the coupling of local regulatory feedback and resulting in maintenance of persistent dysregulated activity states.

### 5.3. Cortex-mediated withdrawal

Motivation, emotion, and cognitive control are severely disrupted during SUD withdrawal and are regulated by the prefrontal cortex (PFC) and the anterior cingulate cortex (ACC) (Goldstein & Volkow, 2011; Koob & Volkow, 2010). Here we synthesize findings from the PFC and ACC regions in humans, and the analogous infralimbic cortex, prelimbic cortex, and ACC in rodents. Together, all regions are referred to as the “cortex”. Disturbances to cortical regions manifest as impulsivity, stress reactivity, negative affect, reduced motivation for natural rewards, and rigid decision-making that promotes drug use (Anderson et al., 2013; Petrie et al., 2022).

Preclinical models provide mechanistic insight into how glial modulation shapes cortical responses in a sex-dependent manner. Sex-dependent cortical adaptations include excitatory balance, stress reactivity, and metabolic function, potentially arising from glial modulation of cortical circuits. In morphine-withdrawn rats, males show higher PFC glucose metabolism than females, suggesting sex differences in regional brain energy utilization during withdrawal (Santoro et al., 2017). Although glia-neuron metabolic coupling may contribute to this pattern, glial contributions have not been directly investigated. In contrast, females exhibit over three times as much c-Fos expression in the PFC during acute withdrawal from alcohol (24 h), indicating elevated cortical activation compared to males (Li et al., 2019). The strong cravings usually reported by females during early withdrawal, as opposed to the more gradual escalation of craving seen in males, may be explained by this increased cortical activation. Similarly, in cocaine withdrawal, females exhibit *early-onset craving* with modest transcriptional changes (limited Grin1 upregulation), whereas males display *progressive craving* accompanied by broad PFC upregulation of Bdnf-IV, Grin2a, Grin2b, and Grin1 (Towers et al., 2022a). These results suggest that female craving may stem from rapid, short-lived synaptic adaptations, while male craving depends on cumulative glutamatergic remodeling and transcriptional activation in prefrontal circuits.

Trajectories of PFC activity reverse during protracted withdrawal. By 28 days post-alcohol-withdrawal, both sexes exhibit reduced PFC activity below baseline levels (Li et al., 2019). Sex differences in ethanol-related neuronal vulnerability have also been reported, with females exhibiting greater susceptibility to neurotoxic effects than males (Hashimoto and Wiren, 2008; Wilhelm et al., 2015). One explanation for this female susceptibility is that baseline neuroimmune priming in females increases susceptibility to glial energy deficits or inflammatory overload, consistent with mitochondrial stress and cytokine dysregulation seen in ethanol-exposed females (Wilhelm et al., 2016). Additional male-specific vulnerabilities emerge in the context of opioid withdrawal. During opioid withdrawal, males exhibit prolonged cortical hyperexcitability and sustained metabolic activity (Santoro et al., 2017), which may initially preserve excitatory function but eventually results in excitotoxic stress. Inhibition of male PFC activity reduces opioid seeking and withdrawal symptoms (McKendrick et al., 2022).

*Findings from cortical studies point to distinct temporal and mechanistic pathways of cortical adaptation during withdrawal: the female cortex may be more prone to early metabolic or inflammatory collapse, while the male cortex relies on prolonged excitatory and metabolic support that may become maladaptive over time*. Given that glia are key regulators of metabolism, inflammation, and excitatory tone, sex-specific glial responses are likely central to these divergent outcomes.

#### 5.3.1. Astrocytes in cortex-mediated withdrawal

Human studies on the role of cortical astrocytes investigating withdrawal and/or sex differences are limited. Human postmortem studies with broader research questions, however, yield contradictory findings regarding astrocyte reactivity and quantity. While some studies show reduced cortical astrocyte density in individuals with AUD (Guizzetti et al., 2025; Miguel-Hidalgo, 2018), other studies find minimal or inconsistent changes in GFAP expression (Nadler et al., 2025). These differences may be attributed to inconsistencies in how astrocyte reactivity is measured (i.e., GFAP, S100β, ALDH1, morphology, transcriptomics, etc.), pointing to a greater methodological challenge in the field. To establish how astrocytes may impact withdrawal vulnerability in male and female human subjects, future studies will require more detailed cellular and sex-specific analyses of the human cortical astrocytes at different stages of substance use.

Preclinical work has begun to address this gap in human literature and has positioned astrocytes as active regulators of cortical excitability and motivation during withdrawal. In the cortex, astrocytes modulate cognitive flexibility and drug-related behaviors through intracellular signaling and metabolic control (Akter et al., 2023; McKendrick et al., 2022). Chronic ethanol exposure lowers S100β expression in female but not male rats. S100β is critical for regulating cell growth, structure, and metabolism of astrocytes (Brozzi et al., 2009; Michetti et al., 2023). This finding suggests that cortical astrocytes in females are more susceptible to ethanol-related damage, which likely contributes to altered neuronal support and cellular stress management (Wilhelm et al., 2016). Astrocyte dysfunctions seen in response to ethanol may disrupt both metabolic and inflammatory homeostasis within the cortex, thus contributing to the heightened stress sensitivity and affective symptoms observed in females during withdrawal.

Chemogenetic studies add important mechanistic insight but have focused on males. Altering Gi- and Gq-coupled signaling pathways in cortical astrocytes using Designer Receptors Exclusively Activated by Designer Drugs (DREADDs) produces opposing effects on both energy metabolism and behavior. In a rat gambling task, a paradigm that models decision-making under uncertainty with relevance to addictive behaviors, activating Gi signaling in astrocytes lowers extracellular lactate and disrupts mitochondrial biogenesis. Behaviorally, in the same animals, activating Gi signaling in astrocytes results in cognitive inflexibility, blunted reward sensitivity, and poor decision-making. Gq activation does the opposite, enhancing lactate release and improving decision-making (Akter et al., 2024; Akter et al., 2023). Results from chemogenetic studies suggest that changes in cortical astrocyte activity impact motivation and cognitive control, processes that are significantly impacted during withdrawal. Therefore, differences in cortical astrocytic GPCR-mediated or metabolic functions might directly contribute to sex-specific vulnerabilities.

Cortical astrocyte responses to alcohol withdrawal differ by sex at the molecular level. In males, astrocytes cultured from the cortex show reduced GFAP expression following ethanol exposure, consistent with reduced astrocytic activation. This may destabilize synaptic regulation and contribute to short-term hyperexcitability. In males, reduced GLT-1 (EAAT2) expression, elevated glutamine, and decreased dopamine (Das et al., 2016) also suggest a male-specific susceptibility to prolonged excitatory stress, even weeks into withdrawal. Female astrocytes exhibit signs of metabolic and inflammatory dysfunction during ethanol withdrawal, including impaired mitochondrial function, altered cytokine signaling, and reduced glutamate uptake (Wilhelm et al., 2016), which may explain the longer-lasting affective symptoms observed in females. Both males and females also show disruptions to the ubiquitin–proteasome pathway in astrocytes after alcohol exposure; however, consistent with greater inflammatory dysfunction described earlier, changes in cytokine signaling and age-related processes after alcohol exposure are more pronounced in females (Wilhelm et al., 2016). Increased inflammatory changes in females may be explained by sex-specific baseline differences in astrocyte reactivity as described in Section 2.

Functional studies connect changes in astrocytic functions to symptoms and affective outcomes. Blocking GLT-1 in the PFC induces anhedonia (John et al., 2012), and cocaine models show persistent GFAP upregulation weeks into withdrawal, indicating chronic astrocyte reactivity (Bowers and Kalivas, 2003; Webb et al., 2022). However, these experiments were only conducted in males. These results point to astrocyte dysfunction as a potential cause of cortex-mediated sex differences during withdrawal, with *males displaying prolonged hyperexcitability and females displaying greater metabolic and inflammatory exhaustion due to impaired glutamate clearance, mitochondrial instability, and chronic reactivity*.

#### 5.3.2. Microglia in cortex-mediated withdrawal

Transcriptomic studies of human postmortem PFC from individuals with OUD show enrichment for immune activation and changes to the synaptic scaffold. Cell-type deconvolution implicates microglia as major contributors to these pathways (Seney et al., 2021). Though potential sex differences remain unexplored, human postmortem data from individuals with AUD show increased Glut5 and Iba-1 expression in the PFC (He and Crews, 2008), indicating metabolic dysregulation and prolonged microglial activation. Crucially, these results highlight the urgent need for sex-aware, phase-resolved transcriptomic and functional studies to better understand the role of microglial signaling in male and female withdrawal, as both the OUD and AUD cohorts were sex-matched but underpowered for sex-stratified analyses.

Across preclinical models, microglial reactivity emerges as both substance- and sex-dependent. In rats with chronic morphine exposure, activation of PFC microglia drives neuronal hyperexcitability and enhanced synaptic plasticity that promotes pain sensitivity and anxiety-like behavior (Liang et al., 2025). Following chronic ethanol exposure, females mount a pro-inflammatory PFC response associated with greater neurotoxicity, whereas males display a more immunosuppressed, repair-oriented profile (Wilhelm et al., 2014, 2015). This finding further supports the notion that females show heightened cortical inflammation and cell death. In comparison, the cortex in opioid models shows the reverse pattern, with males exhibiting greater proinflammatory cytokine signaling and lower expression of the microglial marker TMEM119 than females (Kumar et al., 2021). Cannabinoid models provide additional nuance, with repeated exposure to the cannabinoid receptor agonist WIN55,212–2 resulting in reduced IBA1 expression in the PFC of male rodents that persisted throughout withdrawal (females were not tested) (Vadas et al., 2025).

Studies of cortical microglia provide evidence contrary to the popular idea that female microglia are always more proinflammatory and functionally reactive. *These data reinforce the need to consider that microglial activation is not only context-responsive but also dependent on unique drugs, withdrawal severity, and sex*. Exaggerated microglial responses may prolong affective and cognitive symptoms, and such responses likely affect how cortical circuits recover after drug cessation. To effectively disentangle microglial contributions to withdrawal, future work must include sex-specific comparisons using integrated microglia profiling to better understand the relationship between microglia structure, gene expression, and function.

#### 5.3.3. Oligodendrocytes in cortex-mediated withdrawal

Clinical research suggests that sex differences are present in myelin-associated proteins in the context of substance withdrawal (Darling & Daniel, 2019; Swamydas et al., 2009), suggesting that myelin plasticity may be differentially compromised during abstinence in males vs. females. Preclinical data support this notion but reveal substance-specific patterns. For instance, in opioid withdrawal, oligodendrocyte lineage programs shift in a sex- and region-specific manner: NG2 and Sox10 expression rise during withdrawal, with females showing greater Sox10 upregulation within the PFC, consistent with female-biased lineage activation (Kumar et al., 2021). Ethanol models display the opposite pattern: although the adult brain continues to generate new oligodendrocytes and myelin, chronic ethanol exposure suppresses myelinogenesis in mixed-sex cohorts, reducing the formation of new sheaths (Guo et al., 2021). However, males show increased myelin-related proteins and oligodendrocyte lineage activation during prolonged ethanol abstinence (Navarro & Mandyam, 2015), while females show little recovery or even persistent myelin loss (Darling and Daniel, 2019). This implies that while females suffer from longer-lasting deficiencies in oligodendrocyte renewal, males may employ a compensatory hypermyelination response following abstinence.

*Collectively, findings imply that existing myelin may be affected uniformly across sexes during drug exposure, but the capacity for myelin repair and remodeling diverges by sex and substance*. Differences in species (rat vs. mouse), exposure routes (drinking vs. vapor), withdrawal duration, and inclusion of both sexes are likely the causes of apparent discrepancies among studies. Functionally, both maladaptive hypermyelination and decreased myelinogenesis may affect network timing by reducing the number of sheaths or by placing them less optimally, which could lead to cognitive and affective impairments during prolonged ethanol withdrawal (Amin et al., 2016). This connection is supported by the fact that PFC demyelination reduces working memory and heightens stress responses (Huang et al., 2024b), highlighting the importance of oligodendrocyte integrity for emotional control. These data emphasize the importance of sex-aware, phase-resolved analyses to disentangle how oligodendrocyte lineage dynamics contribute to cortical dysfunction during substance withdrawal.

#### 5.3.4. Glial interactions in cortex-mediated withdrawal

Various studies provide evidence that cortical reactions to withdrawal are mediated in a sex-specific manner by astrocytes, microglia, and oligodendrocytes. Astrocytes help regulate excitability within cortical circuits by acting on synapses, secreting cytokines, and taking up glutamate. Sometimes those functions bias toward excessive inhibition, and other times toward unstable excitatory drive. These effects are not only dependent on sex, but also on drug and withdrawal severity. By releasing cytokines and altering the extracellular matrix, microglia contribute dynamic neuroimmune functions that affect not only neurons but also astrocytic tone and synaptic stability. Protracted affective and cognitive symptoms may arise when this microglia-astrocyte communication is imbalanced, leading to local inflammation and network instability. Oligodendrocytes contribute on a slower timescale using changes in myelin production and sheath placement to change the speed and synchrony of cortical communication. *Overall, results suggest that a key cause of cortical dysfunction during withdrawal is an unbalanced but coordinated glial network*. Studies that monitor glial gene expression and circuit function longitudinally, including both males and females, are necessary to fully comprehend these interactions.

### 5.4. Hippocampus-mediated withdrawal

The hippocampus is critical for learning, memory, and context-cue associations (Smith and Bulkin, 2014) and has become a central focus in substance use and withdrawal research. Across substances, both structural and metabolic sex differences in the hippocampus are evident. In humans with AUD, males show reduced hippocampal volume compared to females (Topiwala et al., 2017). Females in OUD withdrawal display ultrastructural damage and abnormal activity within hippocampal subfields compared to males in OUD withdrawal (Grace et al., 2021). While these effects may be unique to each substance, the results suggest that male hippocampi undergo macroscopic shrinkage, whereas female hippocampi retain volume but exhibit functionally significant microcircuit disruption. Additionally, several detrimental outcomes, including increased alcohol consumption and relapse, have been linked to these ultrastructural alterations (Grace et al., 2021; Kutlu and Gould, 2016; Mandyam, 2013). Regarding metabolic changes, males exhibit higher hippocampal metabolism than females during opioid withdrawal, particularly in CA3 (Santoro et al., 2017). It is unclear whether this is due to substance-specific mechanisms or disorder stage (active use vs. withdrawal). A structurally diminished but functionally hyperactive hippocampus in males may explain this finding. Clinically, females show stronger cue-provoked craving than males in nicotine and opioid use (Saladin et al., 2012; Yu et al., 2007), suggesting that heightened sensitivity to contextual or emotional cues, potentially arising from altered hippocampal microcircuit activity, may underlie greater relapse vulnerability in females.

Clinical results are supported by preclinical findings, which demonstrate that during drug escalation and withdrawal, males and females show distinct hippocampal subregion involvement. In cocaine models, Fos mapping reveals that activity in the dentate gyrus predicts conditioned place preference (CPP) in males, whereas CA1 and CA3 activity predicts CPP in females (Berry et al., 2024), indicating that distinct subregions contribute to drug-context learning in each sex. In nicotine models, female mice display stronger baseline contextual fear memory than males, measured by freezing when re-exposed to a shock-associated environment (Keady et al., 2024). This suggests that females form contextual associations more robustly under normal conditions, an effect that may translate to stronger emotional or craving responses when encountering drug-associated environments. During nicotine withdrawal, females also show impaired extinction learning and difficulty suppressing these learned associations, which corresponds with elevated hippocampal expression of activity-dependent genes (Keady et al., 2024). Analogously, in a rat model of cocaine abstinence, females exhibit a more rapid intensification of drug-seeking compared to males (Corbett et al., 2021). Acute nicotine exposure enhances contextual fear memory in males but has no effect in females (Keady et al., 2024).

Drug exposure may act as a cognitive enhancer in males, increasing hippocampal plasticity and fortifying drug-context associations that were weaker prior to drug exposure. Associations that were already strong in female rats become even more resistant to extinction during withdrawal. *This suggests there is a drug and sex interaction in the hippocampus: males exhibit increased memory consolidation after drug use, while females are predisposed to strong, extinction-resistant drug-context associations that can easily lead to cue-triggered relapse*.

#### 5.4.1. Astrocytes in hippocampus-mediated withdrawal

Hippocampal astrocytes are instrumental in mechanisms underlying substance use and withdrawal, such as memory formation, plasticity, and synaptic transmission (Abreu et al., 2023; Escalada et al., 2024; Sharma et al., 2023). In human postmortem studies, hippocampal findings paint a complicated and occasionally contradictory picture. According to several studies, drug-dependent subjects exhibit increased hippocampal astrocyte reactivity and hypertrophy compared to controls, as evidenced by increased GFAP fiber elaboration or a higher number of GFAP-positive cells (O’ehmichen et al., 1996; Weber et al., 2013). Other studies, however, have found lower hippocampal astrocyte counts in AUD subjects compared to controls (Korbo, 1999). These disparities are likely caused by variations in substance type and exposure stage, analytical methods (e.g., GFAP immunolabeling versus stereological counting), regional specificity, and the balance between compensatory and degenerative astrocyte states. The generalizability of both these findings is further limited by the fact that the cohorts were either exclusively male or heavily male-skewed. Still, the data point to the possibility that astrocytes in hippocampus-mediated withdrawal might not be consistent. Rather, they are likely to progress from reactive hypertrophy to degenerative phenotypes.

Preclinical research supports this dynamicity by elucidating strong, time- and substance-dependent reactivity of hippocampal astrocytes. Cocaine exposure causes quick but transient astrocytic activation in the dentate gyrus in males: GFAP expression doubles after a single injection, stays elevated after multiple exposures, and then returns to baseline in two weeks (Fattore et al., 2002). Changes in GFAP expression due to cocaine are accompanied by changes in astrocyte number, morphology, and spatial complexity. The effects on females are less studied.

Chronic ethanol exposure has longer-lasting effects than cocaine. The JAK/STAT pathways that control glial reactivity are activated during withdrawal, indicated by increases the expression of GFAP and STAT3 colocalization in the hippocampus (Chen et al., 2021). In contrast to male rats, females exposed to binge-like ethanol show widespread neuronal degeneration and vimentin-positive astrocyte activation, indicative of sustained reactivity that persists across multiple abstinence time points and in multiple hippocampal regions (Guerin et al., 2023). Prolonged astrocyte activation may disrupt local hippocampal signaling and result in affective dysregulation. Indeed, chronic ethanol exposure in female rats produces a pronounced negative affective state and is accompanied by decreased ventral hippocampal excitability (Bach et al., 2021), linking hippocampal dysfunction to affective disturbances in ethanol withdrawal. These results point to the possibility that astrocytes in the female hippocampus are more chronically reactive after ethanol exposure, which could exacerbate neurodegeneration and the development of affective withdrawal symptoms in AUD.

Astrocytes also play a critical role in opioid withdrawal-associated fear learning. In the dorsal hippocampus, heroin withdrawal increases astrocytic surface area and volume within the dentate gyrus, along with greater colocalization between astrocytic membranes and postsynaptic density proteins, evidence of strengthened astrocyte-synapse interactions. These structural changes are accompanied by enhanced withdrawal-related fear learning, which is attenuated by chemogenetic stimulation of astrocytic Gi signaling, establishing a direct link between astrocyte activity and anxiety-like responses during opioid withdrawal (Parekh et al., 2024). However, only male rats were used for these experiments, limiting generalizability.

*These investigations demonstrate that hippocampal astrocytes have distinct sex-specific phenotypes and are highly responsive to addictive substances*. Reactivity markers GFAP, pSTAT3, and vimentin are substantially increased in astrocytes, indicating activation. However, the time course and consequences of this reactivity may differ by drug: cocaine induces only transient morphological changes; ethanol leads to persistent reactivity, a decrease in neurodegeneration, and an increase in anhedonia-associated mechanisms, especially in females; opioids facilitate astrocyte-synapse interactions, which produce maladaptive fear learning. Open questions remain regarding the sex-, time-, and substance-specific attributes of astrocyte involvement in the hippocampus. Answering these questions will assist in understanding relapse vulnerability and the development of astrocyte-targeted interventions.

#### 5.4.2. Microglia in hippocampus-mediated withdrawal

Direct clinical evidence for hippocampal microglial alterations during withdrawal is currently lacking. Preclinical work reveals sex- and drug-specific microglial adaptations. Microglia in the hippocampus exhibit reactivity patterns that are both sex- and drug-specific. In males, short-term ethanol abstinence does not induce a classical reactive phenotype in hippocampal microglia but rather increases the levels of anti-inflammatory cytokines IL-10 and TGF-β (Melbourne et al., 2024). In contrast, microglia in the female hippocampus become more reactive during ethanol abstinence. Exposure to binge-like levels of ethanol results in a greater extent of microglial activation in females but not males, indicating that females may be more vulnerable to subsequent challenge (Barton et al., 2017). Melbourne et al. (2024) showed these female-specific changes are long-lasting; even eighteen days after the last binge, microglial counts and activated morphology remained elevated. This suggests that females display a persistent, reactive microglial state that may differentially contribute to withdrawal symptoms, whereas males show a more transient, anti-inflammatory response that may facilitate recovery or resolution of neuroimmune activation.

Cannabinoid studies show the opposite pattern of microglia reactivity in males, although female mice were not investigated. The use of synthetic cannabinoids (WIN55,212–2 and HU-210) leads to increases in IBA1 and inflammatory markers, but withdrawal decreases IBA1 expression below baseline levels (Vadas et al., 2025). This points to a disruption in hippocampal microglial function during cannabinoid withdrawal, including immune monitoring and synaptic homeostasis. These disruptions may be a factor in the cognitive or affective withdrawal symptoms frequently associated with heavy cannabis use, although females remain to be studied.

Hippocampal microglia also play a role in withdrawal from opioid use. In OUD models, short-term abstinence from fentanyl self-administration robustly decreases multiple cytokines and chemokines in the hippocampus of male rats (IFNγ, IL-2, IL-4, IL-5, IL-12p70, IL17A, IL-18) (Ezeomah et al., 2020); females were not studied. Depletion of microglia does not alter naloxone-precipitated withdrawal behaviors in male or female mice, though it does affect antinociceptive responsivity (El Jordi et al., 2022). Thus, microglia may not affect the recorded somatic symptoms, like jumps and paw tremors, but may instead impact cognitive and affective behaviors that were not tested. Additionally, only acute withdrawal was tested in this paradigm. The absence of female data remains a significant knowledge gap.

*These studies suggest that withdrawal produces drug-specific decreases (cannabinoids, opioids) or delayed activations (alcohol) of proinflammatory microglial responses*. Heterogeneity in response suggests hippocampal microglia integrate both drug- and sex-specific cues to shape inflammatory tone and synaptic restructuring during withdrawal. However, there is comparatively little evidence sufficiently powered to detect sex-differences for hippocampal microglia.

#### 5.4.3. Oligodendrocytes in hippocampus-mediated withdrawal

Human and preclinical studies converge to implicate hippocampal myelin disruption as a lasting consequence of alcohol exposure. In postmortem hippocampus of individuals with AUD, there are decreases in myelination-related gene expression (McClintick et al., 2013), although this study was only conducted in males. These data provide direct evidence of disrupted oligodendrocyte function in human alcohol dependence, but without sex-stratified analyses. Complementary evidence from adolescent binge ethanol (ABET) rodent models demonstrates that alcohol exposure induces long-term behavioral and structural consequences: mice exposed to ABET exhibit persistent anxiogenic behavior and impaired spatial memory, accompanied by persistent decreases in hippocampal myelin density (Rice et al., 2019). However, only male mice were examined in this study. These findings suggest that alcohol exposure induces persistent deficits in hippocampal myelination that may contribute to enduring cognitive and affective deficits seen in AUD withdrawal (Dominguez-Salas et al., 2016; Stevens et al., 2014; Stevens et al., 2015). *Myelin deficiencies may interfere with network synchrony and plasticity, which are essential for learning and drug-cue extinction during abstinence*. Future research should focus on expanding oligodendrocyte investigations to non-ethanol models using both male and female subjects.

#### 5.4.4. Glial interactions in hippocampus-mediated withdrawal

Glial subtypes work together in the hippocampus to control contextual (memory), emotional, and cognitive aspects of withdrawal. Oligodendrocytes, microglia, and astrocytes contribute to large patterns of dysregulation that vary by sex and change across substances. In females, prolonged glial activation is linked with neurodegeneration, emotional dysregulation, and extinction-resistant memory. Astrocyte phenotypes are drug- and dose-dependent. Female microglia exhibit longer activation periods and greater changes in morphology, which may contribute to greater sensitivity to stress and potential relapse. Oligodendrocytes experience chronic myelin deficits during withdrawal, which may indicate a desynchronization of memory-networks during abstinence. *Collectively, these results suggest that glial dysregulation impairs hippocampal plasticity, creating a maladaptive setting where changes in stress responsiveness, contextual memory, and emotional reactivity may drive relapse*. However, more studies are needed to properly elucidate the effect of sex on these vulnerabilities.

### 5.5. NAc-mediated withdrawal

The nucleus accumbens (NAc), a crucial hub for motivation and reward processing, undergoes notable and sex-dependent alterations during withdrawal. Alterations in glutamatergic and GABAergic signaling are common across substances. Human imaging studies show that withdrawal from alcohol and other drugs engages the NAc differently in males and females. In females, withdrawal amplifies excitatory-inhibitory imbalance and recruits broad corticostriatal-limbic networks linked to stress responsivity and relapse vulnerability (Logrip et al., 2018; Potenza et al., 2012). In males, neuroimaging studies reveal reduced NAc activation and lower reward responsivity during early alcohol abstinence, patterns consistent with anhedonia and motivational blunting (Volkow et al., 2010; Wrase et al., 2007; Zijlstra et al., 2009). These clinical differences suggest that relapse risk in females is driven by stress- and cue-reactivity, while in males it may stem from diminished reward sensitivity and impaired motivation.

Female rodent studies complement the human imaging studies, with chronic ethanol exposure and withdrawal disrupting NAc excitatory-inhibitory balance (Chan et al., 2025). Consistent with these circuit-level adaptations, females undergoing nicotine withdrawal show larger dopamine decreases and greater increases in glutamate and GABA compared with males, indicating stronger stress responsivity and disrupted neurotransmission (Carcoba et al., 2018). Structurally, female rodents also have greater dendritic spine density and larger spine heads on medium spiny neurons, especially in the NAc core, suggesting stronger glutamatergic input and connectivity (Forlano and Woolley, 2010).

In males, withdrawal engages the NAc differently. Following extended fentanyl access, male rodents, but not females, develop maladaptive drug preference, even with similar somatic withdrawal symptoms. Methadone treatment normalizes this behavior in males, suggesting that their relapse vulnerability may depend more on μ-opioid receptor signaling within the NAc (Townsend et al., 2021).

The NAc undergoes sex-specific neuroadaptations during withdrawal. Female circuits are particularly sensitive to stress- and cue-related relapse triggers, as evidenced by increased stress responsivity, stronger glutamatergic connectivity, and larger withdrawal-induced shifts in dopamine, glutamate, and GABA. In males, withdrawal appears to promote more persistent alterations in motivational bias toward drug seeking, such as the maladaptive increase in fentanyl choice despite similar somatic withdrawal signs. *These findings highlight that relapse vulnerability emerges through distinct mechanisms across sexes: females may be more prone to relapse in stress- or cue-laden contexts, while males may be more vulnerable to long-term persistence of drug-directed behaviors once dependence has formed*.

#### 5.5.1. Astrocytes in NAc-mediated withdrawal

A primary function of NAc astrocytes is the regulation of glutamate signaling and neuronal glutamate homeostasis. Human research shows that early abstinence alters glutamatergic signaling in the NAc. While other metabolites in alcohol-dependent patients do not change, NAc glutamate levels rise soon after alcohol detoxification and show a positive correlation with craving, suggesting a selective disruption of striatal excitatory balance (Bauer et al., 2013). These results suggest that glutamatergic dysregulation is a major factor contributing to relapse vulnerability. Uncovering how astrocytes contribute to this glutamatergic dysregulation may prove beneficial for designing treatments.

Research on rodents offers a mechanistic understanding of how astrocytes control this glutamate imbalance; however, much of this work has been conducted exclusively in male animals. Astrocytic modulation, via chemogenetic DREADDs or optogenetic activation, regulates glutamate availability. Cue-induced reinstatement causes a spike in glutamate release in the NAc core (Scofield et al., 2016). While increasing astrocytic activity intensifies drug conditioning, inhibiting NAc astrocytes prevents cocaine-conditioned place preference (Mongredien et al., 2025). Consistent with this male-biased literature, astrocytes exhibit robust, experience-dependent transcriptional responses to cocaine that are enriched in reward-related circuits (Holt et al., 2025).

During abstinence, astrocytes also experience structural remodeling, with unique patterns by drug and sex. After long-access cocaine self-administration and 45 days of abstinence, male rats exhibit approximately 40% decreases in astrocyte surface area, volume, and postsynaptic colocalization (Kim et al., 2022; Testen et al., 2018), while female rats do not exhibit alterations (Kim et al., 2022). In addition to this structural remodeling, astrocytes show sex-specific transcriptional profiles in response to cocaine use, indicating encoding of drug experiences in a sex-specific manner, potentially biasing glutamate regulation through fundamentally different molecular pathways (Savell et al.,2020). In ethanol models, abstinence in males increases astrocyte density in the NAc core, which is positively correlated with ethanol motivation (Bull et al., 2014). This elevated density likely reflects greater astrocyte packing or proliferation rather than classic hypertrophic reactivity. The same study established causality: blocking gap-junction hemichannels heightened ethanol motivation, while chemogenetic stimulation of NAc astrocytes suppressed it, although these findings were limited to males. Conversely, studies on females show that the NAc of ethanol-dependent animals has lower GFAP immunoreactivity, suggesting attenuated or blunted astrocytic reactivity (Giacometti et al., 2020).

Astrocyte plasticity in opioid models is more congruent between males and females. In both sexes, heroin cues recruit specific astrocyte subpopulations. Some astrocytes increase proximity to synapses, while others upregulate GLT-1 expression; blocking either subtype enhances cue-induced heroin seeking (Kruyer et al., 2022). Structural studies show that astrocytes respond differently depending on the type of reward. Heroin exposure impairs astrocyte structural plasticity, particularly in the dorsomedial NAc shell, a region linked to extinction and relapse processes (Marini et al., 2025). While astrocytes normally adapt their structure in response to cues, this dorsomedial adaptability is blunted after heroin exposure, suggesting a “locked-in” phenotype that cannot adapt appropriately. However, evidence does not yet show whether astrocytes fail to respond to all cues under prolonged addiction, nor have molecular mechanisms of these “locked-in” states in different NAc zones been fully characterized.

*Findings suggest that astrocytes play a key role in regulating NAc glutamatergic tone, relapse vulnerability, and structural plasticity. While females are resilient to cocaine-induced loss of astrocyte density, males show significant morphological and proliferative adaptations associated with an increased drive to seek drugs*. Sex- and drug-specific patterns of astrocyte signaling are a potentially useful target for restoring glutamatergic tone and likely have consequences for maintaining relapse behavior.

#### 5.5.2. Microglia in NAc-mediated withdrawal

Human studies suggest that NAc microglia contribute to withdrawal-related phenotypes. Individuals with OUD show a transcriptional profile in the NAc that is rich in microglial genes and inflammatory signaling (Seney et al., 2021), suggesting immune activation within reward circuits. However, it is unclear whether microglial responses in the NAc differ between males and females or if these responses contribute to the onset of withdrawal-related affective symptoms.

Across substances, preclinical evidence supports that NAc microglia exhibit both morphological and functional reactivity to drug exposure and withdrawal. For example, in male mice, nicotine withdrawal induces more reactive microglial morphology in the NAc. This is accompanied by elevated pro-inflammatory signaling and anxiety-like behavior, although these effects were only studied in males (Adeluyi et al., 2019). Additional research has revealed that a morphine challenge upregulates TLR4 in NAc microglia (Schwarz and Bilbo, 2013) and that microglia-derived TNF-α mediates cocaine-induced synaptic plasticity in the striatum (Lewitus et al., 2016). Although the experiments were only performed on male mice, these findings support the idea that microglial signaling modulates drug-related behavioral effects.

A more nuanced understanding of NAc microglia is revealed by examining sex differences. While some studies suggest that NAc microglia in males show a more reactive baseline morphology (Linker et al., 2019), stress manipulations reveal sex- and region-specific shifts. Variable stress exposure causes NAc microglia in females to shift toward a more ‘primed’ morphology (larger somas, reduced branching); males show little change at that time point (Tsyglakova et al., 2021). Female mice also exhibit significantly higher protein levels of proinflammatory cytokines and chemokines in the NAc than male mice do during oxycodone withdrawal (Kumar et al., 2021). These findings imply microglia from female NAc may be more sensitized to produce exaggerated inflammatory responses during withdrawal or stress. However, these findings may also be interpreted as a result of increased proinflammatory phenotypes of female microglia at baseline.

Microglial signaling also shapes vulnerability to opioid reinforcement; however, the following studies only investigated male rodents. In male rats, neonatal handling, an early-life stress-buffering intervention, increases anti-inflammatory IL-10 expression in NAc microglia, reduces morphine reward, and attenuates glial activation. Mimicking this effect, direct delivery of IL-10 plasmids into the NAc decreases opioid self-administration without impairing natural reward seeking (Lacagnina et al., 2017). Intra-NAc administration of microglial/glial modulators (e. g., minocycline, p38 inhibitors) reduces the maintenance and reinstatement of morphine-conditioned place preference, further implicating a causal role for microglia (Arezoomandan & Haghparast, 2016; Zhang et al., 2012).

These findings show that microglia in the NAc actively participate in drug-related adaptations. *Preclinical research shows that females exhibit more pro-inflammatory microglia phenotypes during withdrawal, suggesting that elevated inflammatory profiles of NAc microglia facilitate increased sensitivity in the reward system*. Broader immune biases influenced by hormones and gene expression impact microglia's response to challenge and are likely the cause of sex differences. Still, there is a specific research gap regarding the role of microglia in the human NAc during withdrawal, particularly sex-specific phenotypes.

#### 5.5.3. Oligodendrocytes in NAc-mediated withdrawal

A growing body of evidence implicates oligodendrocytes in the NAc as active contributors to substance use pathology. Neuroimaging studies consistently report white matter disruption in individuals with opioid use disorder, particularly across frontostriatal circuits (Bora et al., 2012; Upadhyay et al., 2010), but resolution of changes within the NAc remains limited. Although human data may be limited, preclinical work is beginning to implicate NAc demyelination as a key mechanism underlying withdrawal.

Chronic oxycodone exposure in female rats causes NAc axonal tract deformation, fascicle shrinkage, and decreases in myelin basic protein (MBP) expression, all of which indicate axonal injury and demyelination (Fan et al., 2018). Pro-apoptotic signaling and the integrated stress response are activated concurrently with these structural alterations. Interestingly, pro-apoptotic gene induction in vitro is prevented by pharmacological inhibition of this stress response, suggesting a potential therapeutic target.

At the transcriptional level, opioid effects appear sex specific. In control mice, males exhibit lower Mbp mRNA in the NAc than females, but this difference is abolished after chronic oxycodone withdrawal, suggesting a sex-by-treatment interaction in which females may lose myelin and males may gain it (Kumar et al., 2021). Similarly, NG2 (an OPC marker) increases in males but not females following oxycodone, while Sox10 expression remains higher in males regardless of condition. These patterns imply that males and females may begin with different levels of NAc myelination, but drug exposure may shift both toward an intermediate dysfunctional phenotype. Additionally, single-cell analyses show that morphine causes transcriptional reprogramming in all oligodendrocyte lineage states, from progenitors to mature cells. These studies identified strong changes in oligodendrocyte gene expression, many of which are glucocorticoid receptor (GR) targets (Avey et al., 2018; Skupio et al., 2017), although these studies were only conducted in male mice. This suggests that GR signaling is a crucial mediator of opioid-induced oligodendrocyte adaptation in males.

Ethanol induces a distinct, biphasic pattern of NAc oligodendrocyte remodeling. In both male and female rodents, ethanol initially reduces the number of mature OLs; however, this is eventually countered by an increase that leads to thicker myelin sheaths, upregulated oligodendrocyte differentiation genes, and enhanced NAc–PFC connectivity (Liran et al., 2025). However, this increase in myelination might not be an efficacious compensatory mechanism, as the study also indicated that promyelinating drug clemastine promotes ethanol intake, suggesting that over-myelination in reward circuits may reinforce addictive behavior.

Results collectively show that ethanol and opioids affect NAc oligodendrocyte biology in different ways. Opioids are associated with sex-specific oligodendrocyte phenotypes, with males displaying increased myelination and females displaying decreased myelination and stress-related apoptosis. In contrast, ethanol does not show clear sex differences, but instead produces a time-dependent trajectory, with increased myelin production emerging after an initial loss. *Comparisons between NAc oligodendrocytes in males and females are still rare, but the existing literature suggests females are more prone to opioid-induced myelin loss. The impact of these cellular changes on development of stress sensitivity, cognitive decline, or relapse susceptibility is not well understood. Still, these findings emphasize the need for future examining the effects of sex and withdrawal time course on NAc oligodendrocytes*.

#### 5.5.4. Glia interactions in NAc-mediated withdrawal

*The NAc glial network undergoes coordinated yet drug- and sex-specific changes during withdrawal*. Astrocytes modulate synaptic glutamate and metabolic support, but their reactivity diverges by sex: males often show enhanced astrocytic plasticity, while females display blunted astrocytic responses that may reduce glutamate clearance and increase stress sensitivity. NAc microglia exhibit the opposite pattern: female microglia present a more robust inflammatory response during withdrawal, while male microglia remain at a reactive baseline state. These shifts in microglial tone can further alter astrocyte function and disrupt excitatory-inhibitory balance. Oligodendrocyte changes are more drug-dependent. Opioids cause demyelination and stress-induced apoptosis in females, while male oligodendrocytes increase myelination in a dysregulated manner. Ethanol exposure and subsequent withdrawal generates a biphasic reaction in both males and females, with an initial loss of oligodendrocytes followed by excessive remyelination associated with compulsive drug use. These changes across glial subtypes may explain the sex differences in relapse. Future research should simultaneously investigate sex and withdrawal time course to better understand the nuanced roles of NAc glial plasticity in withdrawal.

## 6. Conclusion - Informing treatments and interventions to prevent relapse

Here, we reviewed all available evidence describing glial cells as both causes and consequences of sex differences in substance use withdrawal. Both clinical and preclinical data support that sex differences in astrocytes, microglia, and oligodendrocytes are key drivers of sex differences in withdrawal symptomology. Evidence suggests that mechanisms underlying these sex differences are specific to the exact drug and brain region being studied. Still, there are several important research gaps for specific cell types, brain regions, and drug types that have yet to be studied (Fig. 4). Closing these research gaps is a vital next step to untangling the mechanisms underlying sex-specific vulnerabilities to withdrawal in clinical populations. Further, it is crucial to understand withdrawal etiologies to better inform interventions and treatments, ultimately preventing relapse and prolonging abstinence.

Understanding relapse vulnerability remains one of the most difficult challenges in addiction research. If we can understand the mechanisms that differentially drive drug-seeking during withdrawal in males vs. females, we can design more efficacious, targeted interventions to prevent relapse. Stress, negative affect, and drug-related cues are among the strongest relapse triggers, but they do not affect all individuals in the same way. Females are more likely to relapse when stress or negative emotions are high, whereas males tend to relapse in social or cue-driven contexts*. These behavioral differences mirror the growing recognition that SUD etiologies give rise to sex-specific vulnerabilities, yet no currently available treatments or interventions address these known sex differences*.

Preclinical work provides mechanistic insight into these clinical patterns: female rodents often show greater drug self-administration, stronger cue associations (craving), and a higher propensity for stress-induced reinstatement than males (Radke et al., 2021). In opioid and stimulant models, ovarian hormones further modulate craving and stress sensitivity (Manvich et al., 2023; Vazquez-Morales et al., 2025). Although data are less clear, some findings suggest sex-specific differences in the protraction and behavioral circumstances of relapse (Anderson & Petrovich, 2017). Still, studies converge on a common theme: stress, emotion, cognition, and memory regulate relapse risk differently in males and females, yet the underlying biology linking behavioral predictors to sex-specific outcomes is undefined. Glial biology, as reviewed here, has been hypothesized as a coordinated cellular network responsible for regulating of these sex differences, but extensive further research is needed to properly characterize the underlying sex-specific mechanisms.

The field is now faced with a clear opportunity: to study substance use withdrawal and subsequent relapse vulnerability through the lens of glial biology. Integrating knowledge across the subfields of glial plasticity, addiction biology, and sex differences will allow us to uncover causal mechanisms driving repeated drug use and will inform treatment designs that stabilize sexually dimorphic circuits rather than simply masking symptoms. Investigating the roles of glia in sex-specific aspects of SUD is essential to ensure that interventions and treatments are efficacious for all individuals. True recovery from addiction depends not only on rewiring neurons but also on restoring the health of the brain’s glial support systems that are left to mediate stress, emotions, cognitions, and memories of addictive behaviors. Future studies of glia as mediators of sex-specific vulnerabilities to substance use will undoubtedly move the field forward and enable a new frontier of sex-specific treatments and interventions for relapse prevention.
